# Identification of fibronectin 1 as a candidate genetic modifier in a *Col4a1* mutant mouse model of Gould syndrome

**DOI:** 10.1242/dmm.048231

**Published:** 2021-04-26

**Authors:** Mao Mao, Tanav Popli, Marion Jeanne, Kendall Hoff, Saunak Sen, Douglas B. Gould

**Affiliations:** 1 Department of Ophthalmology, University of California San Francisco, San Francisco, CA 94143, USA; 2 Department of Epidemiology and Biostatistics, University of California San Francisco, San Francisco, CA 94143, USA; 3 Institute of Human Genetics, University of California San Francisco, San Francisco, CA 94143, USA; 4 Department of Preventive Medicine, University of Tennessee Health Science Center, 66 North Pauline St, Memphis, TN 38163, USA; 5 Department of Anatomy, University of California San Francisco, San Francisco, CA 94143, USA

**Keywords:** Gould syndrome, COL4A1, Basement membrane, Modifier, Fibronectin, Integrin signaling

## Abstract

Collagen type IV alpha 1 and alpha 2 (COL4A1 and COL4A2) are major components of almost all basement membranes. *COL4A1* and *COL4A2* mutations cause a multisystem disorder that can affect any organ but typically involves the cerebral vasculature, eyes, kidneys and skeletal muscles. In recent years, patient advocacy and family support groups have united under the name of Gould syndrome. The manifestations of Gould syndrome are highly variable, and animal studies suggest that allelic heterogeneity and genetic context contribute to the clinical variability. We previously characterized a mouse model of Gould syndrome caused by a *Col4a1* mutation in which the severities of ocular anterior segment dysgenesis (ASD), myopathy and intracerebral hemorrhage (ICH) were dependent on genetic background. Here, we performed a genetic modifier screen to provide insight into the mechanisms contributing to Gould syndrome pathogenesis and identified a single locus [modifier of Gould syndrome 1 (*MoGS1*)] on Chromosome 1 that suppressed ASD. A separate screen showed that the same locus ameliorated myopathy. Interestingly, *MoGS1* had no effect on ICH, suggesting that this phenotype could be mechanistically distinct. We refined the *MoGS1* locus to a 4.3 Mb interval containing 18 protein-coding genes, including *Fn1*, which encodes the extracellular matrix component fibronectin 1. Molecular analysis showed that the *MoGS1* locus increased *Fn1* expression, raising the possibility that suppression is achieved through a compensatory extracellular mechanism. Furthermore, we found evidence of increased integrin-linked kinase levels and focal adhesion kinase phosphorylation in *Col4a1* mutant mice that is partially restored by the *MoGS1* locus, implicating the involvement of integrin signaling. Taken together, our results suggest that tissue-specific mechanistic heterogeneity contributes to the variable expressivity of Gould syndrome and that perturbations in integrin signaling may play a role in ocular and muscular manifestations.

## Abstract (precis)

**Summary:** We performed a genetic screen in *Col4a1* mutant mice to elucidate the mechanisms contributing to Gould syndrome. Our results implicate FN1 as a genetic modifier of some, but not all, aspects of Gould syndrome.

## INTRODUCTION

Collagens are the most abundant proteins in the body, making up ∼30% of the dry weight. The collagen superfamily of extracellular matrix (ECM) molecules comprises 28 members encoded by 46 genes (Ricard-Blum, 2011). Among these, type IV collagens are primordial ECM molecules and are fundamental constituents of specialized structures called basement membranes (Fidler et al., 2017). In mammals, six genes encode the type IV collagens (*Col4a1* to *Col4a6*) and their protein products assemble into three distinct heterotrimers [α1α1α2(IV), α3α4α5(IV) or α5α5α6(IV)]. The α1α1α2(IV) network is ubiquitous throughout development and in most adult tissues and its absence results in embryonic lethality (Pöschl et al., 2004). Pathogenic mammalian *Col4a1* mutations were first identified using forward mutagenesis screens in mice with variable forms of ocular pathology (Favor et al., 2007; Gould et al., 2005; Thaung et al., 2002), and human mutations were identified in individuals with severe inherited or *de novo* porencephaly and early-onset intracerebral hemorrhages (ICHs) (Breedveld et al., 2006; Gould et al., 2005, 2006; Sibon et al., 2007; Vahedi et al., 2007). Studies in humans and mice have subsequently expanded the phenotypic spectrum, and it is now well established that mutations in *COL4A1* and *COL4A2* cause a multisystem syndrome (Jeanne and Gould, 2017; Labelle-Dumais et al., 2019; Mao et al., 2015; Meuwissen et al., 2015; Yoneda et al., 2013; Zagaglia et al., 2018).

This paper marks the first published use of the name Gould syndrome. Over the years, a number of abbreviations have been used to describe the various pathologies associated with *COL4A1* and *COL4A2* mutations, including RATOR, HANAC, PADMAL, BSVD1 and BSVD2 [Online Mendelian Inheritance in Man (OMIM) 1800009, 611773, 618564, 175780 and 614483, respectively]. However, the use of clinically based descriptions is impractical and ineffective for a syndrome for which even affected members within a family can have highly variable involvement of different organs. The use of multiple abbreviations fragments the literature and creates confusion for researchers, physicians and families. In efforts to establish a comprehensive and unified identity, over the past 2 years, family support groups and patient advocacy groups have coalesced on Facebook under the name Gould syndrome, and the name has been used in human interest stories on multiple local news broadcasts. Importantly, a non-profit foundation has been established and is currently enrolling patients in a global registry under the name Gould syndrome (https://www.gouldsyndromefoundation.org), which we have now adopted in response.

Gould syndrome is highly clinically heterogeneous and can have variable penetrance and severity across many organs. Cerebrovascular, ocular, renal and neuromuscular pathologies are among the most commonly described manifestations. We originally described a mouse model of Gould syndrome with a *Col4a1* splice acceptor mutation that leads to exclusion of exon 41 (Δex41) from the mature transcript and 17 amino acids from the triple-helical domain of the protein (Gould et al., 2005). However, the majority of disease-causing variants reported in humans are missense mutations in highly conserved glycine residues of the triple-helical domain (Jeanne and Gould, 2017). In order to faithfully replicate human disease, we compared the effects of different mutations in an allelic series composed of nine distinct *Col4a1* and *Col4a2* mutant mouse strains (Favor et al., 2007; Jeanne et al., 2015; Kuo et al., 2014). We established that allelic heterogeneity has important implications for penetrance and severity of various pathologies including ICH and myopathy. We found that mutations can differ in degree or kind, and that allelic differences contribute to clinical variability, in part, by tissue-specific mechanistic heterogeneity (Labelle-Dumais et al., 2019). However, variable clinical manifestations including age of onset and severity among organs have been reported in people with the same recurrent or inherited mutations, and reduced penetrance and asymptomatic carriers exist in some families, indicating the involvement of factors in addition to allelic heterogeneity (Coupry et al., 2010; de Vries et al., 2009; Rødahl et al., 2013; Shah et al., 2012).

Studies using inbred strains of mice clearly demonstrate that a mutation can have variable outcomes in different genetic contexts, underscoring the important role that genetic modification plays in clinical heterogeneity. When compared to other mutations in the allelic series, *Col4a1^Δex41^* tends to have the most severe phenotypes across multiple organs when maintained on a pure C57BL/6J (B6) genetic background (Gould et al., 2007; Jeanne et al., 2015; Kuo et al., 2014). However, when the *Col4a1^+/Δex41^* B6 mice were crossed for a single generation to the CAST/EiJ (CAST) genetic background, ocular anterior segment dysgenesis (ASD), ICH and skeletal myopathy were all significantly reduced in the F_1_ progeny (CASTB6F1) (Gould et al., 2007; Jeanne et al., 2015; Labelle-Dumais et al., 2011). Interestingly, when *Col4a1^+/Δex41^* B6 mice were crossed for a single generation to the 129SvEvTac (129) genetic background, ASD was ameliorated (albeit to a lesser extent than in CASTB6F1 mice) but ICH was not (Gould et al., 2007; Jeanne et al., 2015). Collectively, these data suggest that the B6 genetic background confers susceptibility to develop severe Gould syndrome-related pathologies and that CAST and 129 strains have one or more locus/loci that can genetically suppress pathology in a tissue-specific manner.

Identification of genetic modifier genes can help reveal disease mechanisms and potential therapeutic targets. Phenotype-driven mapping studies using mice and other model organisms are a powerful approach to identify genetic interactions that may be difficult to discover in humans even when large pedigrees are available (Ceco and McNally, 2013; Meyer and Anderson, 2017; Vieira et al., 2015). We previously performed a genetic modifier screen for pathology caused by the *Col4a1^Δex41^* mutation to provide insight into the underlying pathogenic mechanisms. Because (1) ASD is relatively severe, easily screened and robustly suppressed by two strains, and (2) the magnitude and breadth of phenotypic rescue was greater in CAST compared to 129 mice, we selected ASD and CAST as the phenotype and strain, respectively, to perform a pilot suppressor screen in a small number of mice (Gould et al., 2007). We generated CASTB6F1 mice and iteratively crossed mutant mice back to the B6 background. In each backcross, we applied selective pressure to retain one or more genetic suppresser locus by choosing the progeny with the mildest phenotype to breed for the next generation. Using a crude genome-wide scan, we identified a dominant modifier locus in CAST Chromosome (Chr) 1 that suppressed ASD. However, the approach had significant limitations, including screening on a single phenotype, the inability to identify potential recessive loci and the possibility that we may have incidentally overlooked other dominant loci by imposing bottlenecks at each generation.

Here, we performed independent genetic modifier screens for ASD and skeletal myopathy on a large-scale F_2_ cross with the potential to identify multiple dominant or recessive loci that enhanced or suppressed pathology. Both screens identified a single suppressor locus on CAST Chr 1 that we called modifier of Gould syndrome 1 (*MoGS1*). Surprisingly, although the CAST background also suppressed ICH, the effect was not attributable to the *MoGS1* locus. These data suggest that ASD and skeletal myopathy may be mechanistically related to each other but distinct from ICH, which further supports the notion of tissue-specific mechanistic heterogeneity contributing to the clinical variability of Gould syndrome (Labelle-Dumais et al., 2019). Furthermore, molecular analyses suggest that the ECM protein fibronectin 1 (FN1) is a strong candidate as the genetic suppressor at the *MoGS1* locus and provide evidence for a role of altered integrin signaling in Gould syndrome pathogenesis.

## RESULTS

### High-resolution genome-wide mapping identified a suppressor locus for *Col4a1*-related ASD and myopathy on mouse Chr 1

**Fig. 1.**
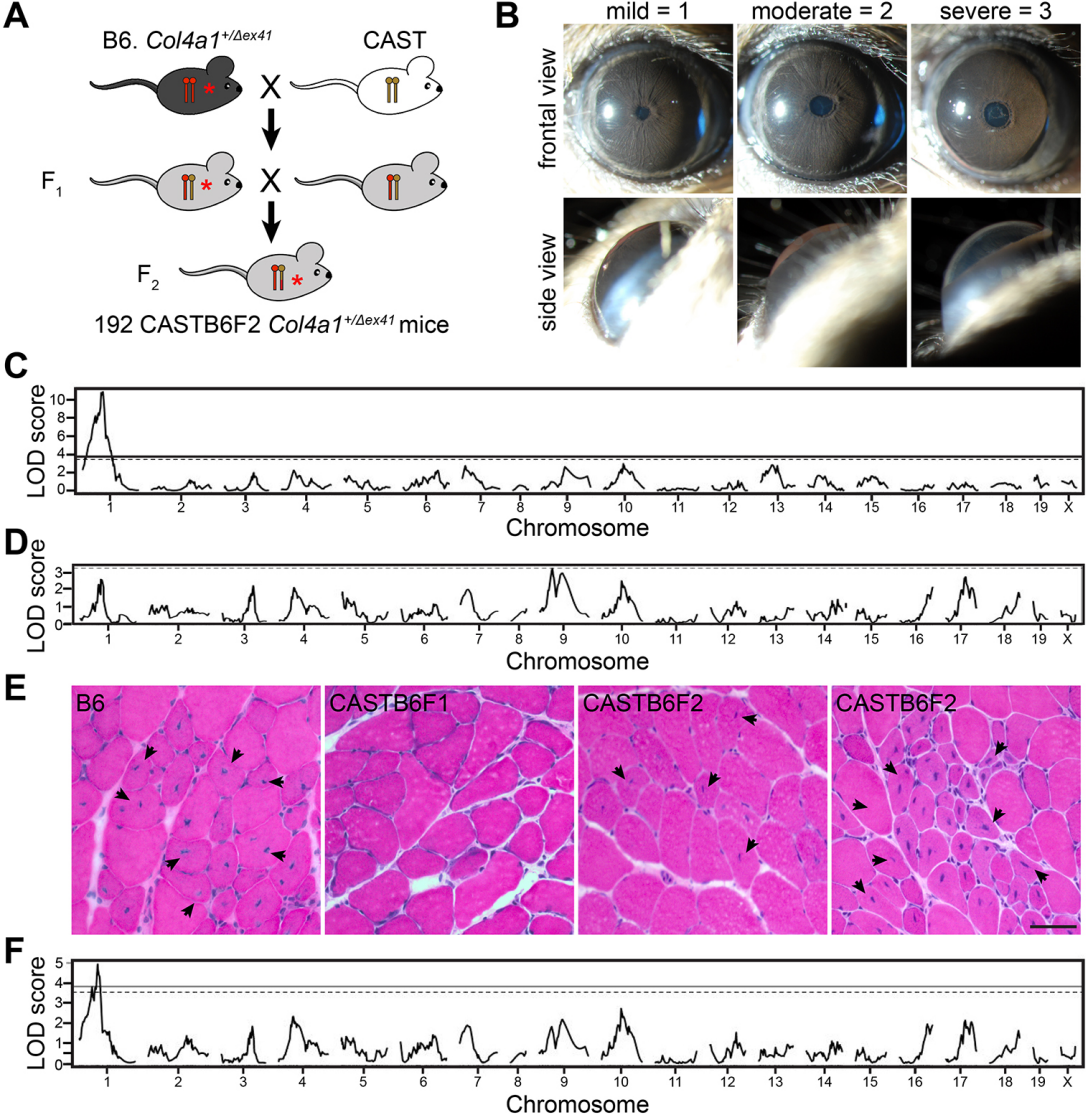
**A genome-wide screen identified a single modifier locus on Chr 1 for anterior segment dysgenesis (ASD) and myopathy.** (A) Schematic representation of the (CAST X B6) F_2_ cross; 192 mutant (CAST X B6-*Col4a1^+/Δex41^*) F_2_ progeny were genotyped and phenotyped for ASD. (B) Representative slit-lamp images illustrating the scoring of ASD severity: mild, 1; moderate, 2; severe, 3. (C) A one-dimensional genome scan identified a locus on Chr 1 for ASD (max. LOD score=11.2, 99% Bayesian confidence interval, extending from 51.3 to 73.0 Mb, Ensembl GRCm38.p6). Solid horizontal line, 5% false positive threshold; dashed horizontal line, 10% threshold. (D) A genome-wide scan conditioned on the Chr 1 modifier locus suggests that no other single locus has a strong effect on ASD. Dashed horizontal line, 20% false positive threshold. (E) Representative images of cross sections from quadriceps stained with H&E, showing variable myopathy severity in *Col4a1^+/Δex41^* mice with different genetic backgrounds. Arrows indicate muscle fibers with non-peripheral nuclei. Scale bar: 20 μm. (F) To identify skeletal muscle modifier loci, a subset (49) of the 192 (CAST X B6) F_2_ progeny was assessed for myopathy. One-dimensional genome scan identified a myopathy modifier at the same position as the ASD modifier (max. LOD score=5.61).

To identify modifiers of Gould syndrome, we first performed a genome-wide screen with the early-onset and easily observed ASD phenotype. We phenotyped and genotyped 192 mutant F_2_ progeny from (CAST X B6-*Col4a1^+/Δex41^*) F_1_ intercrosses using a mouse medium-density linkage panel with 646 informative single-nucleotide polymorphisms (SNPs) (Fig. 1A-C). We identified a region of interest on CAST Chr 1 with a logarithm of the odds (LOD) score of 11.2 and Bayesian confidence interval extending from 51.3 to 73.0 Mb (Fig. 1C). To determine whether smaller effects of other loci might have been masked, we performed a second genome-wide scan conditioned on this locus, but no additional loci reached statistical significance (Fig. 1D). This observation suggests that the major modifying effect of the CAST background on ASD is imparted through this single dominant locus on Chr 1.

In addition to suppressing ASD, the CAST genetic background significantly suppresses skeletal myopathy in *Col4a1* mutant mice (Labelle-Dumais et al., 2011). To identify potential modifier loci for this phenotype, we performed an independent genome-wide mapping analysis using skeletal myopathy as the primary phenotype. We quantified myopathy severity as the percentage of muscle fibers containing non-peripheral nuclei (NPN) in histological sections of quadriceps (Fig. 1E). This assay is more quantitative with greater statistical power but also more labor intensive, and therefore we analyzed a randomly generated subset (*n*=49) of the mutant (CAST X B6-*Col4a1^+/Δex41^*) F_2_ progeny. The genome-wide scan revealed an interval on Chr 1 defined by the same markers as the ASD modifier locus (max. LOD score=5.61) (Fig. 1F).

**Fig. 2.**
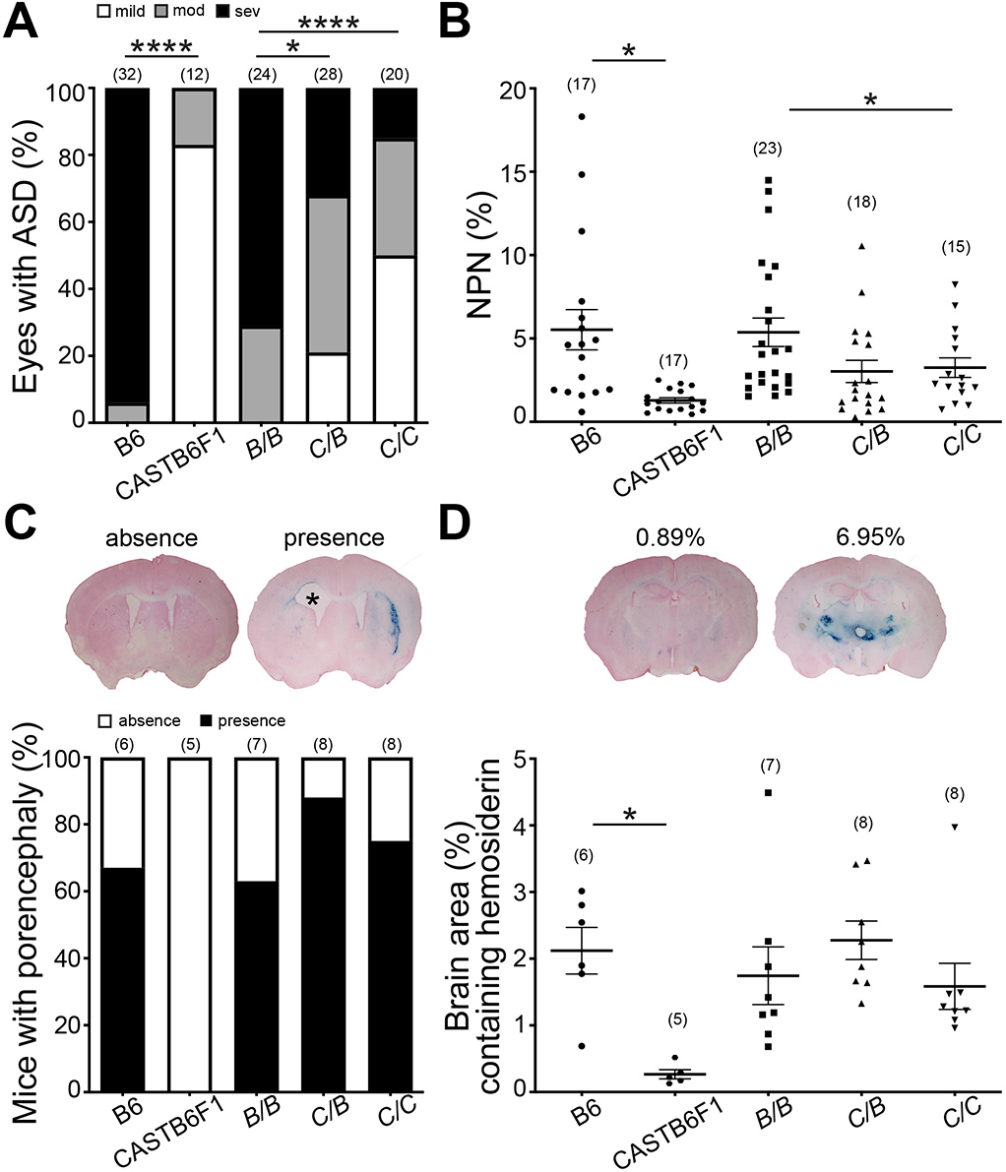
**An incipient congenic strain containing the CAST-derived *MoGS1* locus suppressed ASD and myopathy, but not intracerebral hemorrhage (ICH).** (A) Evaluation of eyes from the incipient congenic mice at N_5_F_2_ validates *MoGS1* as a genetic suppressor of ASD. The bar graph shows the percentage of eyes at each ASD severity level from *Col4a1^+/Δex41^* mice that are homozygous for B6 alleles (B/B), heterozygous or homozygous for CAST alleles (C/B and C/C, respectively) in the congenic interval. ASD for *Col4a1^+/Δex41^* mice on the pure B6 and CASTB6F1 backgrounds is also shown. *P<0.05, ****P<0.0001, by Kruskal–Wallis test followed by Dunn's multiple comparison test. (B) Evaluation of muscles from the N_5_F_2_ congenic mice validates *MoGS1* as a genetic suppressor of skeletal myopathy. Data are presented as mean±s.e.m. *P<0.05, by one-way ANOVA followed by Tukey's multiple comparison test. (C) Penetrance of porencephaly for *Col4a1^+/Δex41^* mice with different genetic backgrounds was determined by brain histology. Top representative images show absence or presence of porencephaly. The asterisk marks the brain area with porencephaly. (D) ICH in mice was assessed using Perl's Prussian Blue staining. Data for each mouse are shown as a percentage of brain area with Prussian Blue staining averaged over 28 sections from each brain. Representative images of Prussian Blue-stained brain sections and respective Prussian Blue staining quantification are shown above the graph. Sample sizes are indicated in parentheses. Data are presented as mean±s.e.m. *P<0.05, unpaired Student's *t*-test for comparison between B6 and CASTB6F1; one-way ANOVA followed by Tukey's test for multiple comparisons between B/B, C/B and C/C.

To validate the biological effect of the *MoGS1* locus, we independently generated an incipient congenic strain by iterative backcrossing the CAST-derived locus onto the susceptible B6 genetic background for five generations (N5). At N5, the genetic background of the mice is ∼97% pure B6 on average, except that they carry the CAST Chr 1 interval that includes the *MoGS1* locus. Incipient congenic mice were intercrossed to generate *Col4a1^+/Δex41^* mice with zero (B/B), one (C/B) or two (C/C) copies of the CAST-derived chromosomal interval (Fig. 2). Of 24 eyes from *Col4a1^+/Δex41^* mice that were homozygous for the B6 allele (*MoGS1^B/B^*) at the congenic interval, 29% (7) were moderate, and 71% (17) were severe (Fig. 2A). In contrast, of 28 eyes from the heterozygous group (*MoGS1^C/B^*), 21.5% (6), 46.5% (13) and 32% (9) of the eyes were scored with mild, moderate and severe ASD, respectively. In mice homozygous for the CAST allele (*MoGS1^C/C^*), 50% (10), 35% (7) and 15% (3) of the eyes were mild, moderate and severe, respectively. In a parallel experiment, we tested the effect of this congenic locus on myopathy and observed a dosage effect, whereby *Col4a1^+/Δex41^* mice that were heterozygous at the *MoGS1* locus (*MoGS1^C/B^*) showed a trend toward reduced myopathy, while homozygous *MoGS1^C/C^* incipient congenic mice had significantly milder myopathy compared to mice that were homozygous for the B6 allele (*MoGS1^B/B^*). Together, these results support the existence of one or more semi-dominant modifier genes at the *MoGS1* locus on Chr 1.

### *MoGS1* does not reduce porencephaly penetrance or ICH severity in *Col4a1* mutant mice

Individuals with Gould syndrome have highly penetrant and clinically variable cerebrovascular diseases that include porencephaly and ICH (Bilguvar et al., 2009; de Vries et al., 2009; Giorgio et al., 2015; Vilain et al., 2002). ICH was previously reported to be significantly reduced in *Col4a1* mutant mice on a CASTB6F1 background compared to those maintained on a B6 background, suggesting that the CAST genetic background also suppresses this phenotype (Jeanne et al., 2015). To test whether the *MoGS1* locus might also genetically modify cerebrovascular diseases, we assessed porencephaly penetrance and ICH severity of the incipient congenic mice. We found that porencephaly penetrance and ICH severity were similar among *Col4a1^+/Δex41^* mice, irrespective of their genotypes at the *MoGS1* locus (Fig. 2C,D).

### Fine mapping with subcongenic lines refined *MoGS1* to a 4.3 Mb interval

**Fig. 3.**
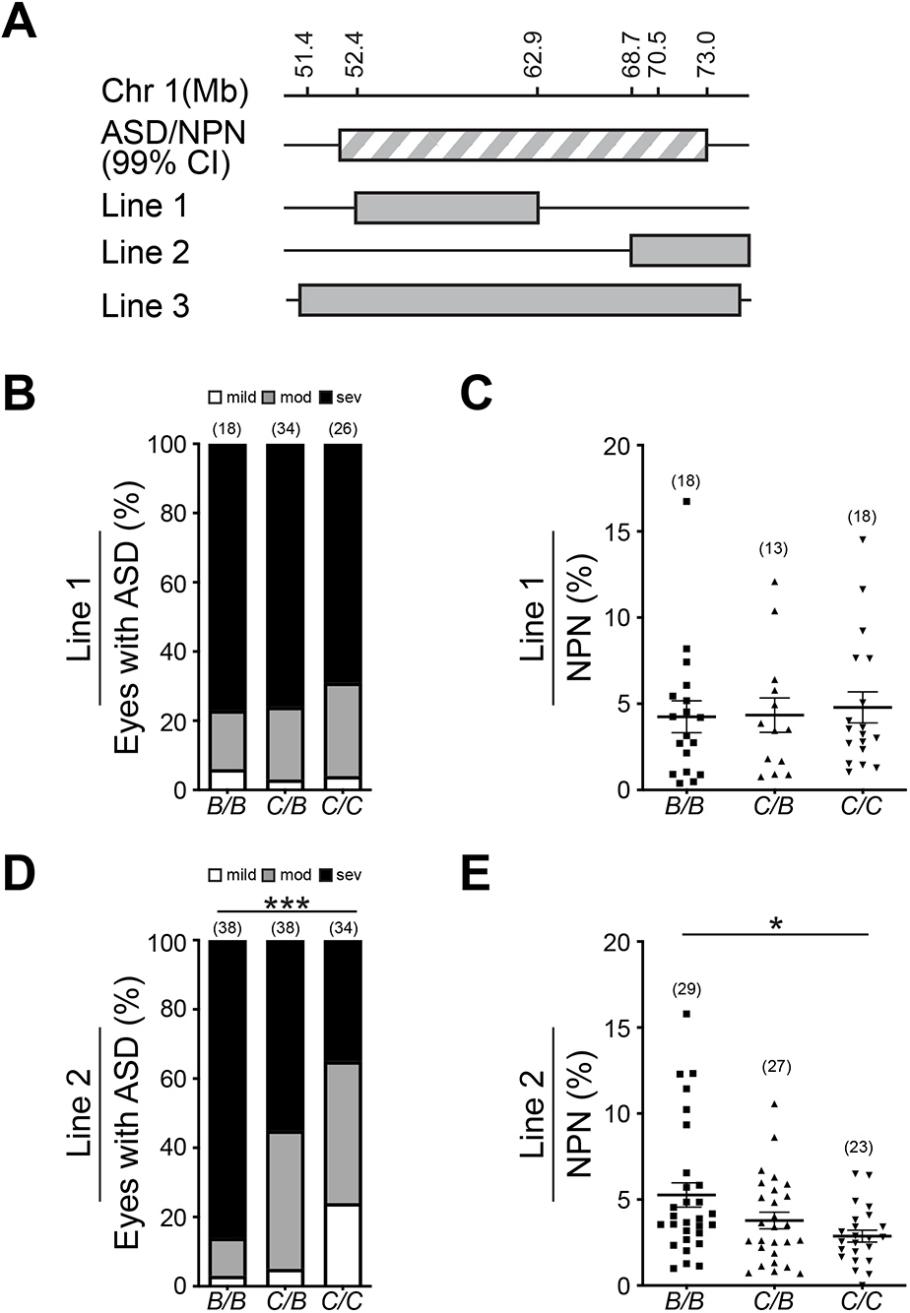
**Fine mapping of the *MoGS1* locus.** (A) Schematic illustration of subcongenic lines containing different intervals of the CAST modifier locus backcrossed onto the B6 background for five generations. Grey-white striped bar indicates the chromosomal location of the full length MoGS1 locus. Black lines, chromosome fragments from B6; gray bars, chromosome fragments from CAST. CI, critical interval. (B,C) A ∼10 Mb proximal portion of the CAST *MoGS1* locus (Line 1, 52,418,578-61,808,145 bp on Chr 1, Ensembl GRCm38.p6) showed no protective effect for ASD (B) or skeletal myopathy (C) phenotypes seen in *Col4a1^+/Δex41^* mice. (D,E) Subcongenic Line 2 containing a distal portion of the CAST *MoGS1* locus (68,723,089-72,973,981 bp on Chr 1, Ensembl GRCm38.p6) ameliorated ASD (D) and myopathy severity (E). *P<0.05, ***P<0.001 by Kruskal–Wallis test followed by Dunn's multiple comparison test in B and D or by one-way ANOVA followed by Tukey's multiple comparison test in C and E. Sample sizes are indicated in parentheses. Data are presented as mean±s.e.m. in C and E.

To refine the interval of interest at the *MoGS1* locus, we generated subcongenic lines of the CAST-derived chromosomal fragments on a B6 background (N5) and tested which line suppresses ASD and skeletal myopathy in *Col4a1^+/Δex41^* mice (Fig. 3A). Line 1, which included an interval of ∼10 Mb from the CAST genome, did not have a modifying effect on either phenotype (Fig. 3B,C). In contrast, a subcongenic line (Line 2) containing the distal portion of the *MoGS1* locus (68.7-73.0 Mb) showed a significant protective effect for both ASD (Fig. 3D) and myopathy (Fig. 3E). Although ∼87% of eyes from *Col4a1^+/Δex41^;MoGS1^B/B^* mice had severe ASD (1 mild, 4 moderate and 33 severe), only ∼35% of the eyes from *Col4a1^+/Δex41^;MoGS1*^C/C^ mice were severe (8 mild, 14 moderate and 12 severe). Likewise, *Col4a1^+/Δex41^;MoGS1^C/C^* mice had significantly fewer NPN compared to *Col4a1^+/Δex41^;MoGS1^B/B^* mice. Heterozygosity for this distal interval had intermediate effects for both phenotypes.

### Candidate gene analysis

The refined *MoGS1* locus is 4.3 Mb and contains 18 protein-coding genes and 38 predicted non-protein coding genes, including 13 long non-coding RNAs (lncRNAs), six small nuclear RNA (snRNA) genes, pseudogenes and others (Table S1). Using publicly available databases (Keane et al., 2011), we examined the refined *MoGS1* interval for sequence differences between the B6 and CAST genomes. Among all variations, there were 40,237 SNPs, 6579 small insertions and deletions (indels), and 166 large structural variations including large insertions or deletions. Thirty-five SNPs were predicted to be missense variants, affecting ten protein-coding genes (Table 1). Among those genes, *Ankar* had three missense variants predicted to be deleterious by Sorting Intolerant From Tolerant (SIFT; Vaser et al., 2016) and phyloP (Pollard et al., 2010), and *Spag16* had one predicted stop-gain variant and one missense variant that was predicted to be damaging by SIFT. However, neither gene is an obvious functional candidate. In addition, *Ikzf2*, *Atic*, *Xrcc5* and *Smarcal1* each have one SNP predicted to be damaging by either phyloP or PROVEAN (Choi and Chan, 2015) (Table S2). None of these SNPs in the *MoGS1* locus was consistently predicted to be damaging by all three tools. Based on the Gene Expression Database (GXD) from Mouse Genome Informatics (informatics.jax.org/expression), 13 of the 18 protein coding genes have been shown to be expressed in eyes and in skeletal muscles. Two genes, *Vwc2l* and *Bard1*, are only expressed in eyes, and three genes, *Ankar*, *Spag16* and *Abca12*, have no reported expression in either tissue. Because both ocular and muscular defects in *Col4a1^+/Δex41^* mice were suppressed by the *MoGS1* locus, we hypothesized that the underlying pathogenic mechanism(s) may be shared between these two tissues. Therefore, despite the presence of possible pathogenic variants, we excluded *Ankar*, *Spag16*, *Abca12*, *Vwc2I* and *Bard1* from further analysis.

**Table 1.**
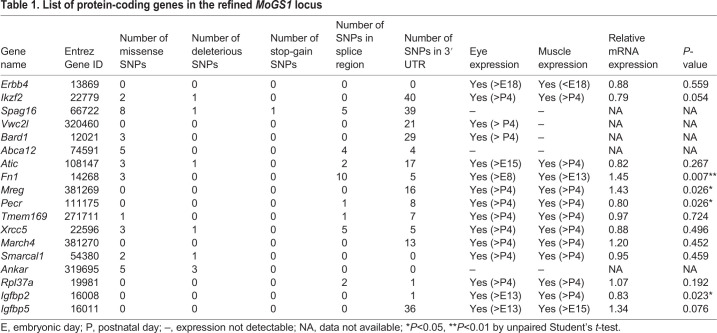
List of protein-coding genes in the refined *MoGS1* locus

**Fig. 4.**
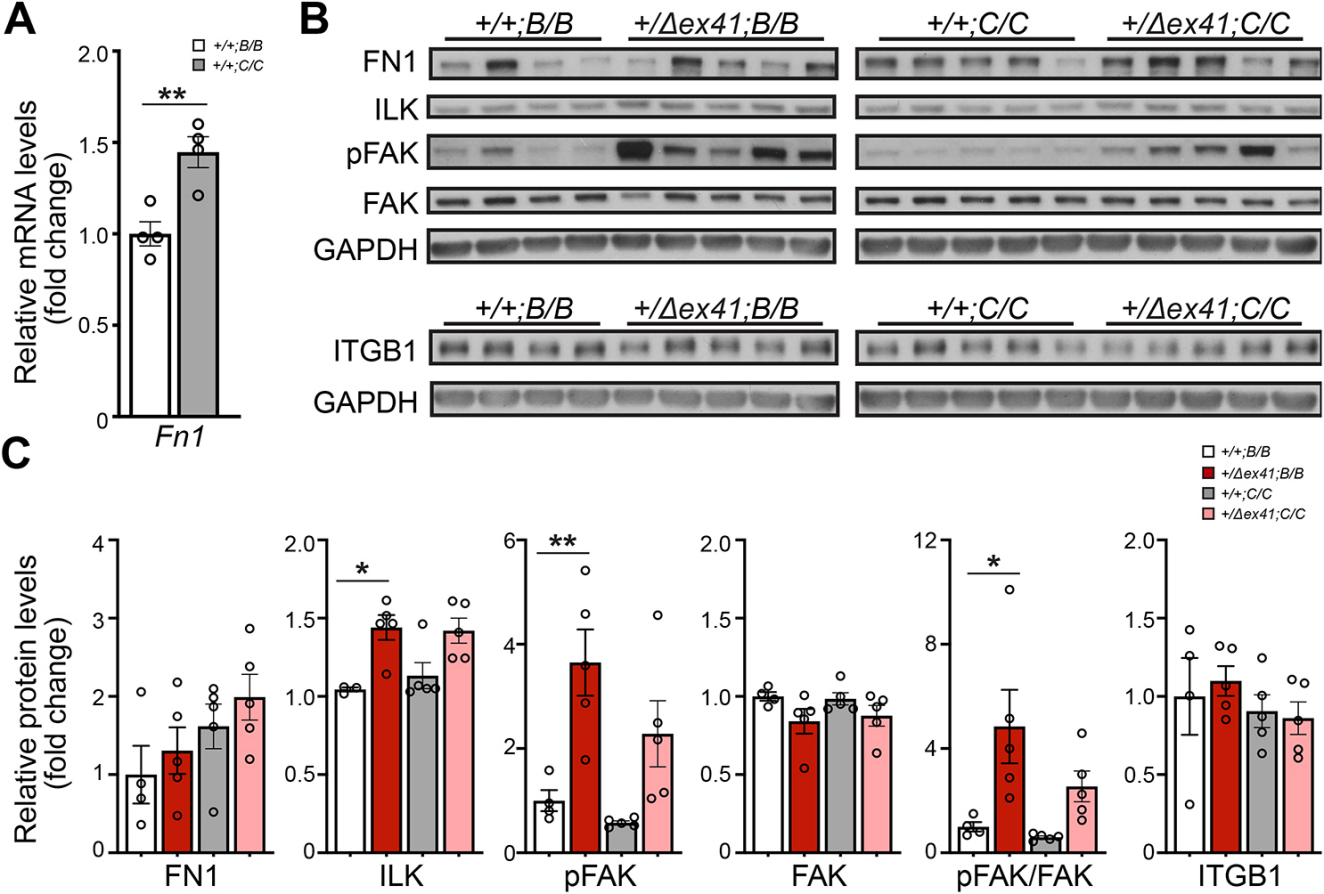
**Evaluating FN1 as a candidate modifier gene.** (A) qPCR analysis showed increased expression of *Fn1* mRNA in P0 eyes from *Col4a1^+/+^* mice homozygous for the CAST interval at the refined *MoGS1* locus (*+/+;C/C*) compared to mice homozygous for the B6 interval (*+/+;B/B*). *n*=4 per genotype. Data are presented as mean±s.e.m. by unpaired Student's *t*-test. (B,C) Representative images (B) and quantification (C) of western blot analyses of P10 quadriceps, showing a trend towards increased FN1 protein levels in mice homozygous for CAST alleles at the refined *MoGS1* locus (*+/+;C/C* and *+/Δex41;C/C*) compared to mice homozygous for B6 alleles (*+/+;B/B* and *+/Δex41;B/B*). ILK levels were increased in *Col4a1^+/Δex41^* mice irrespective of the genotype at the *MoGS1* locus (*+/Δex41;B/B* and *+/Δex41;C/C*, *P*=0.06 for *MoGS1^C/C^*) compared to their corresponding *Col4a1^+/+^* controls (*+/+;B/B* and *+/+;C/C*, respectively). Ratios of pFAK/FAK were significantly elevated in *Col4a1^+/Δex41^* mice homozygous for *MoGS1* B6 alleles (*+/Δex41;B/B*) but not CAST alleles (*+/Δex41;C/C*) (*P*=0.35) compared to their corresponding *Col4a1^+/+^* controls (*+/+;B/B* and *+/+;C/C*, respectively), suggesting altered integrin signaling that is partially restored by the *MoGS1* locus*.* Levels of ITBG1 did not differ between genotypes. *n*=4 *+/+;B/B*, *n*=5 *+/Δex41;B/B*, *n*=5 *+/+;C/C* and *n*=5 *+/Δex41;C/C.* Data are presented as mean±s.e.m. *P<0.05, **P<0.01 by one-way ANOVA followed by Sidak's multiple comparison test.

Next, we tested whether any of the 13 genes with reported expression in ocular and muscular tissues are differentially expressed in B6 mice with or without the CAST interval at the *MoGS1* locus. Four genes, *Mreg*, *Fn1*, *Pecr1* and *Igbp2*, showed significantly increased expression in *MoGS1^C/C^* eyes at postnatal day (P)0 compared to *MoGS1^B/B^* eyes by quantitative PCR (qPCR) (Table 1 and Fig. 4A). FN1 is an ECM protein with multiple important roles in development and tissue homeostasis by interacting with cell surface receptors, ECM proteins and growth factors (Zollinger and Smith, 2017). Notably, FN1 has binding sites for type IV collagen (Laurie et al., 1986, 1982) and localizes adjacent to basement membranes in many tissues (Laurie et al., 1982). Moreover, like collagens, FN1 interacts with cells via integrin receptors (Johansson et al., 1997; Vandenberg et al., 1991), making it a strong functional candidate as a genetic modifier of COL4A1-related pathology.

### Functional testing of FN1 and integrin signaling in *MoGS1* mice

*In silico* analysis comparing the CAST and B6 alleles of *Fn1* revealed three missense SNPs, ten splice region SNPs, 13 SNPs in the UTR and 639 other non-coding SNPs, and 135 small indels and two structural variations within the locus (Table S3). Murine FN1 has 12 isoforms (White et al., 2008), making it difficult to predict the functional consequence(s) of a particular variant. The primary consequence of *Col4a1* mutations is impaired secretion of mutant α1α1α2(IV) heterotrimers into the basement membranes (Kuo et al., 2014) and it is possible that increased FN1 confers partial compensation for extracellular α1α1α2(IV) deficiency. Consistent with this hypothesis, we observed increased *Fn1* expression in developing eyes from *MoGS1^C/C^* mice compared to *MoGS1^B/B^* mice (Fig. 4A). Furthermore, we found that FN1 levels were higher in P10 quadriceps from *Col4a1^+/Δex41^* compared to *Col4a1^+/+^* mice and in *MoGS1^C/C^* compared to *MoGS1^B/B^* mice (Fig. 4B,C). FN1 plays important roles in cell adhesion, migration and signaling during tissue morphogenesis, which is primarily mediated through its interaction with integrins (Miyamoto et al., 1998; Sakai et al., 2003). Therefore, we tested the effect of *MoGS1* on integrin signaling by evaluating the protein levels and phosphorylation status of two downstream effectors, integrin linked kinase (ILK) and focal adhesion kinase (FAK; also known as PTK2) (Hu and Luo, 2013; Humphries et al., 2019) (Fig. 4B,C). Western blot analysis of P10 quadriceps revealed that ILK levels were higher in *Col4a1^+/Δex41^* mice irrespective of whether they carried the *MoGS1^B/B^* or *MoGS1^C/C^* interval. Similarly, FAK phosphorylation (pFAK) and pFAK/FAK ratio were significantly higher in *Col4a1^+/Δex41^;MoGS1^B/B^* compared to *Col4a1^+/+^;MoGS1^B/B^* mice, and were reduced by the *MoGS1^C/C^* interval. No difference was detected for the broadly used beta subunit of the integrin receptor, ITGB1, between genotypes. Taken together, these data suggest that elevated integrin signaling may contribute to Gould syndrome and that ASD and myopathy may be partially rescued by compensatory *Fn1* expression.

## DISCUSSION

Here, we performed a genetic modifier screen in *Col4a1* mutant mice to gain insight into the pathogenic mechanisms that contribute to Gould syndrome. To this end, we used a large-scale F_2_ cross of CAST×B6 and conducted independent screens for ASD and skeletal myopathy – two phenotypes that are commonly observed in individuals with Gould syndrome. Independent analyses for both phenotypes revealed a single, shared, semi-dominant locus on CAST Chr 1 that corroborates a previously identified locus (Gould et al., 2007). To validate these genetic data, we iteratively backcrossed the locus onto the B6 background and showed that a refined interval, referred to as *MoGS1*, suppressed ASD and myopathy in *Col4a1* mutant mice. However, the *MoGS1* locus had no effect on porencephaly penetrance and ICH severity – two phenotypes associated with Gould syndrome and previously characterized in *Col4a1* mutant mice. The failure of *MoGS1* to suppress ICH severity was unexpected because the CASTB6F1 genetic context significantly suppresses this phenotype. These data suggest that the effect of the *MoGS1* locus is tissue specific and that other modifiers of ICH exist in the CAST background.

Using subcongenic strains, we refined the minimum critical interval for the suppressor locus to a 4.3 Mb region containing 18 protein-coding genes. Of the 18 positional candidate genes, 13 are expressed both in eyes and muscles, of which six contained missense variants between the CAST and B6 genomes; however, none were predicted to be deleterious by at least two of the three software prediction tools. Mutations in one of these six genes, *Smarcal1*, causes a rare multisystem disorder (Schimke Immunoosseous dysplasia; OMIM 242900) characterized by spondyloepiphyseal dysplasia, nephrotic syndrome and T-cell immunodeficiency, and a portion of patients develop corneal opacity, myopia, astigmatism and optic atrophy (Boerkoel et al., 2000). SWI/SNF-related matrix-associated, actin-dependent regulator of chromatin, subfamily a-like 1 (SMARCAL1) is a chromatin-remodeling protein involved in transcriptional regulation and DNA replication, repair and recombination (Bansbach et al., 2010). Compared to the B6 reference genome, CAST has two missense variations in *Smarcal1*. One was predicted to be tolerated by all three tools and the other was predicted to be damaging in one transcript isoform by PROVEAN, and no change of expression was detected in P0 eyes. Of the four positional candidate genes that were differentially expressed, *Fn1* is a strong functional candidate. FN1 is a ubiquitously expressed extracellular glycoprotein that plays important roles in multiple processes by interacting with cell surface receptors, growth factors and other ECM proteins. In cell culture, FN1 promotes collagen IV deposition, assembly and incorporation into the ECM, and rescued some cellular phenotypes caused by a *COL4A2* mutation (Filla et al., 2017; Ngandu Mpoyi et al., 2020). Mice deficient in *Fn1* have aberrant lens placode formation and develop microphthalmia and cataracts (Hayes et al., 2012; Huang et al., 2011). Moreover, lack of FN1 in the skeletal muscle stem cell niche impairs muscle regeneration in aged mice (Lukjanenko et al., 2016). Collectively, these findings support our genetic and molecular evidence suggesting that FN1 is a strong candidate as the gene responsible for the effects conferred by the *MoGS1* locus.

In general, the presence of mutant COL4A1 or COL4A2 leads to intracellular accumulation of mutant heterotrimers at the expense of their secretion. Intracellular heterotrimer accumulation represents a potential cell-autonomous (proximal) insult (Jeanne and Gould, 2017; Mao et al., 2015). Impaired heterotrimer secretion can also lead to a cell non-autonomous (distal) insult caused by extracellular heterotrimer deficiency, which can simultaneously perturb any number of presently unidentified cellular pathways. Intracellular accumulation and extracellular deficiency can be addressed simultaneously by targeting the proximal defect of protein misfolding. For example, pharmacologically promoting heterotrimer secretion using a chemical chaperone, 4-phenylbutyrate (4PBA), alleviates diverse pathologies in *Col4a1* mutant mice (Hayashi et al., 2018; Jeanne et al., 2015; Jones et al., 2019; Labelle-Dumais et al., 2019). However, even in the absence of 4PBA, secretion of mutant heterotrimers is not completely abolished, and the presence of mutant heterotrimers in the basement membrane represents a third and distinct class of insult. We demonstrated this proof of concept by showing that mice with a *Col4a1^G394V^* mutation have disproportionately severe myopathy, which is exacerbated when they are treated with 4PBA (Labelle-Dumais et al., 2019). The affected residue, glycine 394, is adjacent to a putative integrin-binding domain (Parkin et al., 2011), implicating impaired integrin binding in skeletal myopathy. Thus, for mutations that impair subdomains of the heterotrimer responsible for executing specific extracellular functions, promoting secretion of mutant heterotrimers would still be predicted to ameliorate many extracellular functions. However, the increased levels of mutant heterotrimers in basement membranes may also exacerbate specific disease pathway(s) related to the function(s) of the impacted subdomain (Labelle-Dumais et al., 2019). Identifying the multitude of extracellular roles of COL4A1/2 and how they are executed will be key in understanding fundamental aspects of matrix biology and the tissue-specific pathogenic mechanisms in Gould syndrome. Mapping functional subdomains on the α1α1α2(IV) heterotrimers will be important for genetically stratifying patients and may influence the prognosis and potential therapeutic approaches.

The unbiased nature of genetic screens leaves open the possibility for finding modifiers of either proximal or distal insults. By independently analyzing two phenotypes using an inbred strain (CAST) with broad phenotypic suppression, we sought to find modifiers that might represent therapeutic targets for the breadth of Gould syndrome phenotypes. *MoGS1*, the only significant locus that we identified, conferred tissue-specific effects by suppressing ASD and myopathy but not ICH. This observation is consistent with a modifier that has an extracellular function and suggests that the two phenotypes have at least partially overlapping distal pathogenic mechanisms. In a previous study using the *Col4a1^Δex41^* mutation, we found that, compared to B6, the CASTB6F1 background appeared to reduce intracellular accumulation but did not increase extracellular COL4A1 levels (Jeanne et al., 2015). This observation implicated intracellular accumulation in pathogenesis but did not rule out the potential of compensation by other extracellular factors. The failure of *MoGS1* to suppress ICH severity suggests a mechanism independent of *Fn1* expression levels, and intracellular accumulation of mutant proteins may indeed be the primary pathogenic insult for that phenotype. Further experiments are required to determine the molecular basis for the suppression of ICH severity.

When we compared disease severity across a murine allelic series of nine *Col4a1* and *Col4a2* mutations, we found that the *Col4a1^G394V^* mutation had relatively little intracellular accumulation and that *Col4a1^+/G394V^* mice had mild ICH but severe ASD and myopathy (Kuo et al., 2014). The proximity of the *Col4a1^G394V^* mutation to a predicted integrin-binding domain suggests that impaired integrin binding contributes to ocular dysgenesis and myopathy. Therefore, one possibility is that FN1 ameliorates ASD and skeletal myopathy caused by *Col4a1* mutations through compensatory integrin-mediated interactions. Consistent with this hypothesis, we detected evidence for increased integrin signaling in *Col4a1^+/Δex41^* mice that was reduced by the *MoGS1* locus from CAST in the context of increased *Fn1* expression. Moreover, ILK and FAK are both previously implicated in lens development (Cammas et al., 2012; Harburger and Calderwood, 2009; Hayes et al., 2012; Samuelsson et al., 2007; Teo et al., 2014) and myopathy (Boppart and Mahmassani, 2019; Gheyara et al., 2007). Although we detected increased *Fn1* expression, this does not preclude the potential contributions of alternative splicing. Understanding the precise mechanism of action and functionally validating this pathway with experimental manipulation and preclinical interventions would represent a significant advance in the understanding of the pathogenic mechanisms that contribute to Gould syndrome.

## MATERIALS AND METHODS

### Mice

All experiments were compliant with the ARVO Statement for the Use of Animals in Ophthalmic and Vision Research and approved by the Institutional Animal Care and Use Committee at the University of California San Francisco (UCSF). *Col4a1^+/Δex41^* mice were originally identified in a mutagenesis screen conducted at The Jackson Laboratory (Bar Harbor, ME, USA) (Gould et al., 2007, 2005). Since then, *Col4a1^+/Δex41^* mice have been backcrossed to B6 for at least 20 generations. CAST mice were obtained from The Jackson Laboratory. All animals were maintained in full-barrier facilities free of specific pathogens on a 12-h light/dark cycle with food and water *ad libitum*. Both male and female mice were used in this study.

### Slit-lamp biomicroscopy

Ocular anterior segment examinations were performed on mice at 1.0-1.5 months of age using a slit-lamp biomicroscope (Topcon SL-D7; Topcon Medical Systems, Oakland, NJ, USA) attached to a digital SLR camera (Nikon D200; Nikon, Melville, NY, USA).

### Muscle analysis

At 2 months of age, mice were subjected to treadmill exercise and their quadriceps were harvested 2 days later. Exercise was 30 min with a 15° downhill grade on a treadmill equipped with a shock plate (Columbus Instruments, Columbus, OH, USA). Animals were started at 6 m/min and increased by 3 m/min every 2 min until a maximum of 15 m/min speed was reached. Quadriceps were dissected and frozen in liquid nitrogen-cooled isopentane. Cryosections (10 μm) were collected at regular intervals and stained with Hematoxylin and Eosin (H&E) for histopathology. Then, the number of NPN were evaluated on a total of 12 sections for each muscle, and the percentage of muscle fibers with NPN was quantified. The observers were masked to genotypes and counted between 2000 and 5000 muscle fibers per animal.

### Brain histological analysis

ICH was assessed at 2 months of age by Perl's Prussian Blue staining as previously described (Jeanne et al., 2015). Briefly, mice underwent transcardial perfusion with saline followed by 4% paraformaldehyde (PFA) and then were fixed in 4% PFA overnight. Brains were dissected, cryoprotected in 30% sucrose and embedded in optimal cutting temperature compound (Sakura Finetek, Torrance, CA, USA). Coronal cryosections (35 μm) regularly spaced along the rostro-caudal axis were stained with Prussian Blue/Fast Red and imaged. On each section, the percentage brain area with Prussian Blue staining was calculated using ImageJ software (National Institutes of Health). ICH severity was expressed as the average percentage of hemosiderin surface area on 28 sections for each brain. The presence or absence of porencephaly on sections used in ICH analysis was also recorded.

### Genome scan

Genomic DNA were obtained from 192 (CAST X B6) F_2_ mice carrying the *Col4a1^+/Δex41^* mutation and genotyped at the UCSF Genomic Core Facility with a commercial SNP panel (Illumina, San Diego, CA, USA), which contains 646 informative SNPs for (CAST X B6) F_2_ progeny. A genome-wide suppressor screen was performed in R (R Core Team, 2019) using the package R/qtl (Broman et al., 2003), treating the trait as binary. Genome-wide significance was established using 1000 permutation testing (Churchill and Doerge, 1994). A confidence interval for each quantitative trait locus (QTL) location was then calculated using Bayesian confidence sets (Sen and Churchill, 2001). The ASD score for each animal was averaged from both eyes and treated as a dichotomous variable; a score of 1 was mild, and a score of 1.5-3 was severe. A subset (49) of these 192 mice was randomly selected in a separate mapping analysis for the muscle modifier as a quantitative trait.

### *In silico* analysis

Variants between B6 and CAST were exported from the Sanger Mouse Genome Project website (Keane et al., 2011; Yalcin et al., 2011) and annotated using the Ensembl Genome Browser (Hunt et al., 2018; Yates et al., 2020). SNPs with high and moderate levels of impact were prioritized. Non-synonymous SNPs were further analyzed using phyloP (Pollard et al., 2010), PROVEAN (Choi and Chan, 2015), in addition to SIFT (Ng and Henikoff, 2003), a built-in feature in Ensembl. PhyloP scores more than 1, PROVEAN scores less than −2.5 and SIFT scores less than 0.05 were considered to be likely damaging.

### qPCR

Eyes from P0 mice were dissected in PBS, immediately transferred in RNA*later* (ThermoFisher Scientific, USA) and stored at −80°C until use. Total RNA was extracted using TRIzol Reagent (ThermoFisher Scientific) according to the manufacturer's instructions and reverse transcribed to complementary DNA (cDNA) using an iScript cDNA synthesis kit (Bio-Rad, Hercules, CA, USA). qPCR was performed on a Bio-Rad CFX96 real-time system using SsoFast Evagreen mix (Bio-Rad) and primers are listed in Table S4. Briefly, 10 ng cDNA and 1.25 μM primers were used per reaction in a final volume of 10 μl. Each cycle consisted of denaturation at 95°C for 5 s, followed by annealing and extension at 60°C for 5 s, for a total of 45 cycles. All experiments were run with technical duplicates and four to six biological replicates were used per group. The relative expression of each gene was normalized to *Hprt1* or *Gapdh* and analyzed using the 2^−ΔΔCT^ method (Livak and Schmittgen, 2001).

### Western blot analyses

Quadriceps from P10 pups were dissected and total proteins extracted using radioimmunoprecipitation assay (RIPA) buffer (VWR, Radnor, PA, USA) supplemented with Halt Protease and Phosphatase Inhibitor Cocktail (ThermoFisher Scientific), EDTA and 2 mM phenylmethylsulfonyl fluoride. Total proteins (10 μg) were separated on 4-15% gradient SDS-PAGE gels (Bio-Rad) and transferred to polyvinylidene fluoride (PVDF) membranes (Bio-Rad). Membranes were blocked for 1 h at room temperature in 5% non-fat milk in TBS containing 0.1% Tween-20 (TBST), incubated overnight at 4°C in primary antibodies diluted in 2% non-fat milk in TBST. Primary antibodies and dilutions used are as follows: rabbit anti-FN1 (1:10,000; Abcam, ab2413), rabbit anti-pFAK (1:1000; Invitrogen, 44624G), rabbit anti-FAK (1:1000; Santa Cruz Biotechnology, sc-557), rabbit anti-ILK (1:1000; Cell Signaling Technology, 3862), mouse anti-ITGB1 (1:1000; BD Biosciences, 610467) and mouse anti-GAPDH (1:500,000; Millipore, MAB374). Following washes in TBST, membranes were incubated for 1 h at room temperature with species-specific horseradish peroxidase-conjugated secondary antibodies (1:10,000; Jackson ImmunoResearch, West Grove, PA, USA) diluted in 2% non-fat milk in TBST. Immunoreactivity was visualized using chemiluminescence (ECL or Luminata Forte substrate, ThermoFisher Scientific) and imaged using a ChemiDoc MP Imaging System (Bio-Rad) or exposed to X-ray films. Densitometric analyses were performed on low-exposure images using the Quantity One software (Bio-Rad). To compare samples loaded on different gels, all samples were normalized to the same calibrator sample (a wild-type B6 sample) that was loaded on all gels.

### Statistics

Statistical analyses and graphs were prepared using Prism v8.0 software (GraphPad, La Jolla, CA, USA). Multiple comparisons were carried out using one-way ANOVA followed by Tukey post-test or Kruskal–Wallis test followed by Dunn's post-test. *P*-values less than 0.05 were considered statistically significant.

## Supplementary Material

Supplementary information
