# Supplementary material for: Identification of fibronectin 1 as a candidate genetic modifier in a Col4a1 mutant mouse model of Gould syndrome
Source: Dis Model Mech. 2021 Apr 26;14(4):dmm048231. doi: 10.1242/dmm.048231 (PMC8106953; doi:10.1242/dmm.048231)
Supplement: Supplementary information [file dmm-14-048231-s1.pdf]

Table S1. List of protein-coding and non-coding genes in the *ModGS1* locus.

**Interval**	**Marker**	**Coordinates (bp on Chr1) in Mouse Build38 (Ensembl GRCm38.p6)**
99% confidence interval-start	rs13475866	51,398,699
95% confidence interval-start	rs6353774	59,723,178
99% or 95% confidence interval-end	rs13475919	72,973,981
MoGS1 -start	D1Mit178	68,723,089
MoGS1 -end	D1Mit383	82,778,395

search results from Enzembl (1: 68723089-72973981bp) by biomart)

Gene stable ID	Strand	Gene start (bp)	Gene end (bp)	Gene description	Gene name	Gene type
ENSMUSG00000062209	-1	68032186	69108059	erb-b2 receptor tyrosine kinase 4 [Source:MGI Symbol;Acc:MGI:104771]	Erbb4	protein_coding
ENSMUSG00000025997	-1	69531214	69687245	IKAROS family zinc finger 2 [Source:MGI Symbol;Acc:MGI:1342541]	Ikzf2	protein_coding
ENSMUSG00000053153	1	69826970	70725132	sperm associated antigen 16 [Source:MGI Symbol;Acc:MGI:1913972]	Spag16	protein_coding
ENSMUSG00000045648	1	70725715	70885397	von Willebrand factor C domain-containing protein 2-like [Source:MGI Symbol;Acc:MGI:2444069]	Vwc2l	protein_coding
ENSMUSG00000026196	-1	71027498	71103146	BRCA1 associated RING domain 1 [Source:MGI Symbol;Acc:MGI:1328361]	Bard1	protein_coding
ENSMUSG00000050296	-1	71242276	71414910	ATP-binding cassette, sub-family A (ABC1), member 12 [Source:MGI Symbol;Acc:MGI:2676312]	Abca12	protein_coding
ENSMUSG00000026192	1	71557150	71579631	5-aminoimidazole-4-carboxamide ribonucleotide formyltransferase/IMP cyclohydrolase [Source:MGI Symbol;Acc:MGI:1351352]	Atic	protein_coding
ENSMUSG00000026193	-1	71585520	71653200	fibronectin 1 [Source:MGI Symbol;Acc:MGI:95566]	Fn1	protein_coding
ENSMUSG00000039395	-1	72159442	72212307	melanoregulin [Source:MGI Symbol;Acc:MGI:2151839]	Mreg	protein_coding
ENSMUSG00000026189	-1	72259167	72284314	peroxisomal trans-2-enoyl-CoA reductase [Source:MGI Symbol;Acc:MGI:2148199]	Pecr	protein_coding
ENSMUSG00000026188	1	72284369	72303104	transmembrane protein 169 [Source:MGI Symbol;Acc:MGI:2442781]	Tmem169	protein_coding
ENSMUSG00000026187	1	72307427	72394952	X-ray repair complementing defective repair in Chinese hamster cells 5 [Source:MGI Symbol;Acc:MGI:104517]	Xrcc5	protein_coding
ENSMUSG00000039372	-1	72427112	72536930	membrane-associated ring finger (C3HC4) 4 [Source:MGI Symbol;Acc:MGI:2683550]	March4	protein_coding
ENSMUSG00000039354	1	72583251	72633134	SWI/SNF related matrix associated, actin dependent regulator of chromatin, subfamily a-like 1 [Source:MGI Symbol;Acc:MGI:1859183]	Smarcal1	protein_coding
ENSMUSG00000039342	-1	72642980	72700579	ankyrin and armadillo repeat containing [Source:MGI Symbol;Acc:MGI:2442559]	Ankar	protein_coding
ENSMUSG00000046330	1	72711290	72713813	ribosomal protein L37a [Source:MGI Symbol;Acc:MGI:98068]	Rpl37a	protein_coding
ENSMUSG00000039323	1	72824503	72852474	insulin-like growth factor binding protein 2 [Source:MGI Symbol;Acc:MGI:96437]	Igfbp2	protein_coding
ENSMUSG00000026185	-1	72857932	72874884	insulin-like growth factor binding protein 5 [Source:MGI Symbol;Acc:MGI:96440]	Igfbp5	protein_coding
ENSMUSG00000087514	-1	69091913	69106740	predicted gene 16076 [Source:MGI Symbol;Acc:MGI:3802084]	Gm16076	lncRNA
ENSMUSG00000099804	1	69441056	69444084	predicted gene 28497 [Source:MGI Symbol;Acc:MGI:5579203]	Gm28497	lncRNA
ENSMUSG00000100354	-1	69490357	69493788	predicted gene 29113 [Source:MGI Symbol;Acc:MGI:5579819]	Gm29113	lncRNA
ENSMUSG00000101156	1	69513932	69547707	predicted gene 29114 [Source:MGI Symbol;Acc:MGI:5579820]	Gm29114	lncRNA
ENSMUSG00000100815	-1	69595036	69598522	predicted gene 29112 [Source:MGI Symbol;Acc:MGI:5579818]	Gm29112	lncRNA
ENSMUSG00000099954	-1	69660187	69662061	predicted gene 28112 [Source:MGI Symbol;Acc:MGI:5578818]	Gm28112	lncRNA
ENSMUSG00000073652	1	71652837	71662843	apolipoprotein L 7d [Source:MGI Symbol;Acc:MGI:3723449]	Apol7d	lncRNA
ENSMUSG00000099707	1	71888112	71891204	predicted gene 8883 [Source:MGI Symbol;Acc:MGI:3649116]	Gm8883	lncRNA
ENSMUSG00000101211	-1	71894068	71917125	predicted gene 28818 [Source:MGI Symbol;Acc:MGI:5579524]	Gm28818	lncRNA
ENSMUSG00000101848	-1	71958998	72005195	RIKEN cDNA 4933417E11 gene [Source:MGI Symbol;Acc:MGI:1918384]	4933417E11Rik	lncRNA
ENSMUSG00000101764	-1	71983681	72038731	RIKEN cDNA 4930556G22 gene [Source:MGI Symbol;Acc:MGI:1925457]	4930556G22Rik	lncRNA
ENSMUSG00000102123	-1	72107155	72122001	predicted gene 4319 [Source:MGI Symbol;Acc:MGI:3782500]	Gm4319	lncRNA
ENSMUSG00000110712	1	72641188	72642963	predicted gene, 39662 [Source:MGI Symbol;Acc:MGI:5622547]	Gm39662	lncRNA
ENSMUSG00000089345	-1	69921473	69921747	predicted gene, 22665 [Source:MGI Symbol;Acc:MGI:5452442]	Gm22665	misc_RNA
ENSMUSG00000084332	-1	69130914	69131299	predicted gene 8840 [Source:MGI Symbol;Acc:MGI:3647579]	Gm8840	processed_pseudogene
ENSMUSG00000101387	1	69377321	69378071	PC-esterase domain containing 1C, pseudogene [Source:MGI Symbol;Acc:MGI:3644590]	Pced1c-ps	processed_pseudogene
ENSMUSG00000089965	-1	71039652	71040030	predicted gene 16236 [Source:MGI Symbol;Acc:MGI:3801868]	Gm16236	processed_pseudogene
ENSMUSG00000101551	-1	71155246	71156020	predicted gene 8870 [Source:MGI Symbol;Acc:MGI:3645634]	Gm8870	processed_pseudogene
ENSMUSG00000101617	-1	71493737	71493814	predicted gene 6947 [Source:MGI Symbol;Acc:MGI:3647350]	Gm6947	processed_pseudogene
ENSMUSG00000101834	-1	71709705	71710055	predicted gene 5829 [Source:MGI Symbol;Acc:MGI:3644438]	Gm5829	processed_pseudogene
ENSMUSG00000100319	-1	71714048	71714924	predicted gene 5256 [Source:MGI Symbol;Acc:MGI:3644836]	Gm5256	processed_pseudogene
ENSMUSG00000059483	1	72004288	72004767	predicted gene 5528 [Source:MGI Symbol;Acc:MGI:3643453]	Gm5528	processed_pseudogene
ENSMUSG00000101496	1	72087857	72088454	predicted gene 8885 [Source:MGI Symbol;Acc:MGI:3649121]	Gm8885	processed_pseudogene
ENSMUSG00000085812	1	72459499	72460027	predicted gene 15843 [Source:MGI Symbol;Acc:MGI:3801845]	Gm15843	processed_pseudogene
ENSMUSG00000110487	1	72692864	72693452	predicted gene, 18035 [Source:MGI Symbol;Acc:MGI:5010220]	Gm18035	processed_pseudogene
ENSMUSG00000110596	-1	72706289	72706502	predicted gene 45859 [Source:MGI Symbol;Acc:MGI:5804974]	Gm45859	processed_pseudogene
ENSMUSG00000101730	-1	70067968	70068055	predicted gene 28580 [Source:MGI Symbol;Acc:MGI:5579286]	Gm28580	unprocessed_pseudogene
ENSMUSG00000080524	-1	69955004	69955129	predicted gene, 23879 [Source:MGI Symbol;Acc:MGI:5453656]	Gm23879	snRNA
ENSMUSG00000075952	1	70596815	70596921	predicted gene, 23422 [Source:MGI Symbol;Acc:MGI:5453199]	Gm23422	snRNA
ENSMUSG00000094655	1	72226240	72226430	predicted gene, 25360 [Source:MGI Symbol;Acc:MGI:5455137]	Gm25360	snRNA
ENSMUSG00000094050	1	72236946	72237136	predicted gene, 23472 [Source:MGI Symbol;Acc:MGI:5453249]	Gm23472	snRNA
ENSMUSG00000093956	1	72244213	72244403	predicted gene, 24497 [Source:MGI Symbol;Acc:MGI:5454274]	Gm24497	snRNA
ENSMUSG00000093843	1	72255008	72255198	predicted gene, 25939 [Source:MGI Symbol;Acc:MGI:5455716]	Gm25939	snRNA
ENSMUSG00000103809	-1	68758485	68761727	predicted gene, 37061 [Source:MGI Symbol;Acc:MGI:5610289]	Gm37061	TEC
ENSMUSG00000103402	1	69770868	69772753	predicted gene, 37930 [Source:MGI Symbol;Acc:MGI:5611158]	Gm37930	TEC
ENSMUSG00000103052	-1	70310512	70312911	predicted gene, 38272 [Source:MGI Symbol;Acc:MGI:5611500]	Gm38272	TEC
ENSMUSG00000104287	-1	70571435	70577271	predicted gene, 37456 [Source:MGI Symbol;Acc:MGI:5610684]	Gm37456	TEC
ENSMUSG00000102689	-1	71843090	71846610	predicted gene, 37217 [Source:MGI Symbol;Acc:MGI:5610445]	Gm37217	TEC

Table S2. List of non-synonymous variants in the *MoGS1* locus and functional evaluation
variants likely to be damaging were highlighted in red
'-' data not available

Chr	Position	Gene	dbSNP	Ref	CAST_EiJ	Csq	Transcript	AA	AA coord	SIFT	PolyP	PROVEAN
1	69538980	Ikzf2	rs37128437	T	C	missense_variant	ENSMUST00000027146.8	I/V	457	0.31	**2.58**	0.31
1	69538980	Ikzf2	rs37128437	T	C	missense_variant	ENSMUST00000187184.6	I/V	431	0.38	**2.58**	-
1	69538980	Ikzf2	rs37128437	T	C	missense_variant	ENSMUST00000188110.6	I/V	385	0.42	**2.58**	-
1	69538980	Ikzf2	rs37128437	T	C	missense_variant	ENSMUST00000190771.6	I/V	463	0.35	**2.58**	-
1	69538980	Ikzf2	rs37128437	T	C	missense_variant	ENSMUST00000190855.6	I/V	383	0.36	**2.58**	-
1	69538980	Ikzf2	rs37128437	T	C	missense_variant	ENSMUST00000191262.6	I/V	312	0.33	**2.58**	-
1	69539010	Ikzf2	rs241593732	T	C	missense_variant	ENSMUST00000027146.8	T/A	447	0.82	0.25	-0.96
1	69539010	Ikzf2	rs241593732	T	C	missense_variant	ENSMUST00000187184.6	T/A	421	0.84	0.25	-
1	69539010	Ikzf2	rs241593732	T	C	missense_variant	ENSMUST00000188110.6	T/A	375	0.34	0.25	-
1	69539010	Ikzf2	rs241593732	T	C	missense_variant	ENSMUST00000190771.6	T/A	453	0.75	0.25	-
1	69539010	Ikzf2	rs241593732	T	C	missense_variant	ENSMUST00000190855.6	T/A	373	0.66	0.25	-
1	69539010	Ikzf2	rs241593732	T	C	missense_variant	ENSMUST00000191262.6	T/A	302	0.23	0.25	-
1	69887530	Spag16	rs30450565	G	A	missense_variant	ENSMUST00000065425.11	M/I	261	0.45	0.14	0.21
1	69887530	Spag16	rs30450565	G	A	missense_variant	ENSMUST00000113940.3	M/I	261	1.00	0.14	0.11
1	69896475	Spag16	rs30453095	G	C	missense_variant	ENSMUST00000113940.3	G/A	287	0.27	-1.88	-0.78
1	69896475	Spag16	rs30453095	G	C	missense_variant	ENSMUST00000065425.11	G/A	287	0.54	-1.88	-1.01
1	69896558	Spag16	rs30453099	T	A	missense_variant	ENSMUST00000065425.11	S/T	315	1.00	0.42	0.96
1	69896558	Spag16	rs30453099	T	A	missense_variant	ENSMUST00000113940.3	S/T	315	1.00	0.42	1.20
1	69923728	Spag16	rs231712879	T	A	missense_variant	ENSMUST00000113940.3	S/T	345	0.55	0.48	-0.50
1	69923743	Spag16	rs213985138	C	A	missense_variant	ENSMUST00000113940.3	P/T	350	**0.03**	-0.53	-1.62
1	69923783	Spag16	rs30457410	T	C	missense_variant	ENSMUST00000113940.3	F/S	363	0.51	-0.71	0.23
1	69923729	Spag16	rs258516890	C	A	stop_gained	ENSMUST00000113940.3	**S/***	345	-	0.46	-
1	69923828	Spag16	rs234237412	C	A	missense_variant	ENSMUST00000113940.3	T/K	378	1.00	-1.28	-1.41
1	69923870	Spag16	rs30456524	A	G	missense_variant	ENSMUST00000113940.3	K/R	392	1.00	-0.51	-0.27
1	71047658	Bard1	rs32910349	T	C	missense_variant	ENSMUST00000027393.7	M/V	586	0.15	-1.99	0.06
1	71047762	Bard1	rs32910347	C	G	missense_variant	ENSMUST00000027393.7	G/A	551	0.09	0.50	-0.38
1	71102765	Bard1	rs32908750	C	G	missense_variant	ENSMUST00000027393.7	A/P	15	0.35	-1.38	-0.51
1	71260907	Abca12	rs32899296	A	T	missense_variant	ENSMUST00000087268.6	F/L	2242	0.85	-0.98	1.70
1	71302913	Abca12	rs32895134	A	G	missense_variant	ENSMUST00000087268.6	L/S	963	0.71	-3.05	0.26
1	71303508	Abca12	rs253097417	T	C	missense_variant	ENSMUST00000087268.6	T/A	938	0.84	0.23	-0.08
1	71314144	Abca12	rs217476446	T	G	missense_variant	ENSMUST00000087268.6	N/T	713	0.85	-0.24	-0.51
1	71362617	Abca12	rs260750795	G	T	missense_variant	ENSMUST00000087268.6	P/T	101	0.75	-1.87	0.90
1	71564503	Atic	rs242240822	C	G	missense_variant	ENSMUST00000027384.5	H/Q	159	0.56	**1.04**	1.46
1	71567886	Atic	rs30558118	G	A	missense_variant	ENSMUST00000027384.5	R/K	254	1.00	-0.51	1.43
1	71569049	Atic	rs250986606	G	A	missense_variant	ENSMUST00000027384.5	V/I	301	0.23	0.50	-0.54
1	71597442	Fn1	rs212662229	C	T	missense_variant	ENSMUST00000055226.12	V/I	2184	0.32	-0.04	-0.14
1	71597442	Fn1	rs212662229	C	T	missense_variant	ENSMUST00000186129.6	V/I	2003	0.55	-0.04	-
1	71597442	Fn1	rs212662229	C	T	missense_variant	ENSMUST00000187938.6	V/I	2068	0.26	-0.04	-
1	71597442	Fn1	rs212662229	C	T	missense_variant	ENSMUST00000188894.6	V/I	2093	0.31	-0.04	-
1	71597442	Fn1	rs212662229	C	T	missense_variant	ENSMUST00000190780.6	V/I	1978	0.37	-0.04	-
1	71624203	Fn1	rs30574135	T	C	missense_variant	ENSMUST00000055226.12	N/S	1042	0.33	0.62	-1.20
1	71624203	Fn1	rs30574135	T	C	missense_variant	ENSMUST00000186129.6	N/S	1042	0.38	0.62	-
1	71624203	Fn1	rs30574135	T	C	missense_variant	ENSMUST00000187938.6	N/S	1042	0.45	0.62	-
1	71624203	Fn1	rs30574135	T	C	missense_variant	ENSMUST00000188674.6	N/S	1042	0.33	0.62	-
1	71624203	Fn1	rs30574135	T	C	missense_variant	ENSMUST00000188894.6	N/S	1042	0.48	0.62	-
1	71624203	Fn1	rs30574135	T	C	missense_variant	ENSMUST00000189821.6	N/S	1042	0.28	0.62	-
1	71624203	Fn1	rs30574135	T	C	missense_variant	ENSMUST00000190780.6	N/S	1042	0.38	0.62	-
1	71632044	Fn1	rs30571778	T	C	missense_variant in nmd_transcript	ENSMUST00000186613.1	T/A	71	-	-0.25	-
1	72301070	Tmem169	rs47607413	A	G	missense_variant	ENSMUST00000027380.11	T/A	220	0.96	0.78	0.91
1	72312510	Xrcc5	rs39774306	T	C	missense_variant	ENSMUST00000027379.9	C/R	80	0.17	**1.13**	0.76
1	72312561	Xrcc5	rs36471826	A	G	missense_variant	ENSMUST00000027379.9	K/E	97	0.83	0.49	-0.10
1	72383746	Xrcc5	rs36607208	A	G	missense_variant	ENSMUST00000027379.9	I/V	697	1.00	0.16	0.26
1	72585807	Smarcal1	rs225083018	G	A	missense_variant	ENSMUST00000047615.14	G/S	38	0.19	0.39	-0.84
1	72585807	Smarcal1	rs225083018	G	A	missense_variant	ENSMUST00000133123.7	G/S	38	0.12	0.39	0.06
1	72585807	Smarcal1	rs225083018	G	A	missense_variant	ENSMUST00000145868.7	G/S	38	0.15	0.39	**-2.68**
1	72585807	Smarcal1	rs225083018	G	A	missense_variant	ENSMUST00000152225.1	G/S	38	0.19	0.00	-0.84
1	72586116	Smarcal1	rs33860607	T	C	missense_variant	ENSMUST00000047615.14	W/R	141	0.35	0.00	0.35
1	72586116	Smarcal1	rs33860607	T	C	missense_variant	ENSMUST00000152225.1	W/R	141	0.35	0.39	0.35
1	72651014	Ankar	rs48386843	T	G	missense_variant	ENSMUST00000053499.5	E/D	1219	1.00	0.37	1.97
1	72651014	Ankar	rs48386843	T	G	missense_variant	ENSMUST00000211837.1	E/D	1218	1.00	0.37	-
1	72651014	Ankar	rs48386843	T	G	missense_variant	ENSMUST00000212573.1	E/D	1001	1.00	0.37	-
1	72651015	Ankar	rs231616541	T	C	missense_variant	ENSMUST00000053499.5	E/G	1219	**0.01**	**2.23**	-2.24
1	72651015	Ankar	rs231616541	T	C	missense_variant	ENSMUST00000211837.1	E/G	1218	**0.01**	**2.23**	-
1	72651015	Ankar	rs231616541	T	C	missense_variant	ENSMUST00000212573.1	E/G	1001	**0.01**	**2.23**	-
1	72651031	Ankar	rs51817549	C	T	missense_variant	ENSMUST00000053499.5	A/T	1214	**0.00**	**2.03**	-0.38
1	72651031	Ankar	rs51817549	C	T	missense_variant	ENSMUST00000211837.1	A/T	1213	**0.00**	**2.03**	-
1	72651031	Ankar	rs51817549	C	T	missense_variant	ENSMUST00000212573.1	A/T	996	**0.00**	**2.03**	-
1	72658414	Ankar	rs45924668	T	C	missense_variant	ENSMUST00000053499.5	I/V	1077	0.53	0.54	-0.15
1	72658414	Ankar	rs45924668	T	C	missense_variant	ENSMUST00000211837.1	I/V	1076	0.53	0.54	-
1	72658414	Ankar	rs45924668	T	C	missense_variant	ENSMUST00000212573.1	I/V	859	0.55	0.54	-
1	72687800	Ankar	rs51768524	G	A	missense_variant	ENSMUST00000053499.5	A/V	411	**0.01**	**3.13**	-0.63
1	72687800	Ankar	rs51768524	G	A	missense_variant	ENSMUST00000211837.1	A/V	411	**0.01**	**3.13**	-
1	72687800	Ankar	rs51768524	G	A	missense_variant	ENSMUST00000212573.1	A/V	193	**0.01**	**3.13**	-

Table S3. SNPs, small indels and structural variants in *Fn1*

#Filters used:
Fn1 1:71585520-71653200bp (Ensembl GRCm38.p6)
all variations
Base calling key (first column for each strain):
'A C G T' = High confidence SNP
'a c t g' = Low confidence SNP
'-' (hyphen) = High confidence reference
'~' (tilde) = Low confidence reference
'.' (period) = Genotype not called
'*' (asterisk) = Multiple consequence

Chr	Position	Gene	dbSNP	Ref	CAST_EiJ	Csq
1	71624203	Fn1	rs30574135	T	C*	missense_variant _
1	71597442	Fn1	rs212662229	C	T*	missense_variant intron_variant nmd_transcript_variant downstream_gene_variant _
1	71632044	Fn1	rs30571778	T	C*	missense_variant synonymous_variant nmd_transcript_variant
1	71585895	Fn1	rs30562303	C	T*	3_prime_utr_variant downstream_gene_variant _
1	71586001	Fn1	rs30563076	T	C*	3_prime_utr_variant downstream_gene_variant _
1	71586060	Fn1	rs223714514	A	C*	3_prime_utr_variant downstream_gene_variant _
1	71586075	Fn1	rs246295554	T	C*	3_prime_utr_variant downstream_gene_variant _
1	71600524	Fn1	rs219067116	G	A*	3_prime_utr_variant intron_variant nmd_transcript_variant upstream_gene_variant downstream_gene_variant _
1	71653175	Fn1	rs30575859	T	C*	5_prime_utr_variant intron_variant upstream_gene_variant _
1	71584778	Fn1	rs30561461	A	G	downstream_gene_variant
1	71584882	Fn1	rs30562294	G	C	downstream_gene_variant
1	71585104	Fn1	rs246178380	A	G	downstream_gene_variant
1	71585410	Fn1	rs30562300	A	G	downstream_gene_variant
1	71585512	Fn1	rs211934779	G	A	downstream_gene_variant
1	71608170	Fn1	rs234088824	T	C*	intron_variant _
1	71608172	Fn1	rs243139453	G	A*	intron_variant _
1	71608219	Fn1	rs30569655	C	T*	intron_variant _
1	71608436	Fn1	rs30569658	T	C*	intron_variant _
1	71608510	Fn1	rs30569661	G	A*	intron_variant _
1	71608518	Fn1	rs50239622	C	T*	intron_variant _
1	71608759	Fn1	rs239447297	G	A*	intron_variant _
1	71608763	Fn1	rs256887593	C	G*	intron_variant _
1	71608769	Fn1	rs224943891	T	C*	intron_variant _
1	71608779	Fn1	rs245041957	A	T*	intron_variant _
1	71608832	Fn1	rs266116206	G	T*	intron_variant _
1	71608877	Fn1	rs228380840	C	T*	intron_variant _
1	71608891	Fn1	rs236673973	C	T*	intron_variant _
1	71608933	Fn1	rs253931362	A	G*	intron_variant _
1	71608963	Fn1	rs226173252	C	A*	intron_variant _
1	71609035	Fn1	rs255374465	T	A*	intron_variant _
1	71609048	Fn1	rs216898140	A	T*	intron_variant _
1	71609064	Fn1	rs30570407	A	G*	intron_variant _
1	71609104	Fn1	rs254967350	G	C*	intron_variant _
1	71609328	Fn1	rs30570413	A	G*	intron_variant _
1	71609376	Fn1	rs30571096	C	G*	intron_variant _
1	71614657	Fn1	rs238731161	T	C*	intron_variant _
1	71615004	Fn1	rs234584124	A	T*	intron_variant _
1	71615083	Fn1	rs30574266	T	A*	intron_variant _
1	71615165	Fn1	rs30574269	T	G*	intron_variant _
1	71615179	Fn1	rs30574272	A	G*	intron_variant _
1	71615302	Fn1	rs30575235	T	A*	intron_variant _
1	71615581	Fn1	rs30575241	T	C*	intron_variant _
1	71615615	Fn1	rs246764380	G	A*	intron_variant _
1	71615787	Fn1	rs212333362	T	G*	intron_variant _
1	71615898	Fn1	rs30576084	G	A*	intron_variant _
1	71616089	Fn1	rs252461615	G	C*	intron_variant _
1	71616330	Fn1	rs30576087	G	A*	intron_variant _
1	71616459	Fn1	rs30568375	C	T*	intron_variant _
1	71616507	Fn1	rs30568378	G	A*	intron_variant _
1	71616668	Fn1	rs30568381	T	C*	intron_variant _
1	71616699	Fn1	rs30569284	A	G*	intron_variant _
1	71616745	Fn1	rs250981340	G	A*	intron_variant _
1	71616823	Fn1	rs217495441	G	A*	intron_variant _
1	71616865	Fn1	rs238216717	G	A*	intron_variant _
1	71617023	Fn1	rs217037570	A	G*	intron_variant _
1	71617026	Fn1	rs235247329	C	T*	intron_variant _
1	71617027	Fn1	rs250145790	A	G*	intron_variant _
1	71617029	Fn1	rs220095414	A	T*	intron_variant _
1	71617227	Fn1	rs221959223	A	G*	intron_variant _
1	71617374	Fn1	rs263760770	G	T*	intron_variant _
1	71617656	Fn1	rs261551826	A	G*	intron_variant _
1	71617804	Fn1	rs243260272	C	T*	intron_variant _
1	71617852	Fn1	rs227539934	C	T*	intron_variant _
1	71617856	Fn1	rs260892450	A	G*	intron_variant _
1	71617858	Fn1	rs225324196	C	A*	intron_variant _
1	71617879	Fn1	rs253450011	T	G*	intron_variant _
1	71617945	Fn1	rs250383976	C	T*	intron_variant _
1	71618019	Fn1	rs30569293	G	A*	intron_variant _
1	71618048	Fn1	rs580179531	T	C*	intron_variant _

1	71618199	Fn1	rs230681252	A	T*	intron_variant _
1	71618210	Fn1	rs223401318	A	G*	intron_variant _
1	71618211	Fn1	rs246001700	A	G*	intron_variant _
1	71618274	Fn1	rs30570466	C	T*	intron_variant _
1	71618309	Fn1	rs242974192	T	C*	intron_variant _
1	71618366	Fn1	rs30570472	A	G*	intron_variant _
1	71618517	Fn1	rs30571285	T	C*	intron_variant _
1	71618548	Fn1	rs230494380	G	A*	intron_variant _
1	71618714	Fn1	rs30572174	G	T*	intron_variant _
1	71618739	Fn1	rs30572177	G	A*	intron_variant _
1	71618809	Fn1	rs237069316	C	T*	intron_variant _
1	71618813	Fn1	rs255393758	G	A*	intron_variant _
1	71618863	Fn1	rs587505127	G	A*	intron_variant _
1	71619062	Fn1	rs30572180	C	A*	intron_variant _
1	71619605	Fn1	rs30573106	G	A*	intron_variant _
1	71619752	Fn1	rs215178753	G	C*	intron_variant _
1	71619980	Fn1	rs30573112	T	G*	intron_variant _
1	71620702	Fn1	rs30574934	T	C*	intron_variant _
1	71620711	Fn1	rs215116700	C	T*	intron_variant _
1	71620847	Fn1	rs30574940	C	T*	intron_variant _
1	71620939	Fn1	rs30574943	T	C*	intron_variant _
1	71620947	Fn1	rs258594535	A	C*	intron_variant _
1	71621203	Fn1	rs30576635	T	C*	intron_variant _
1	71621328	Fn1	rs30576641	C	T*	intron_variant _
1	71621442	Fn1	rs234725147	G	A*	intron_variant _
1	71621690	Fn1	rs30568349	A	G*	intron_variant _
1	71621829	Fn1	rs229137892	A	G*	intron_variant _
1	71622150	Fn1	rs30568352	T	C*	intron_variant _
1	71622177	Fn1	rs30569165	C	A*	intron_variant _
1	71622196	Fn1	rs30569168	C	T*	intron_variant _
1	71622265	Fn1	rs30569171	T	C*	intron_variant _
1	71622418	Fn1	rs30570067	A	G*	intron_variant _
1	71622789	Fn1	rs30570073	G	A*	intron_variant _
1	71622881	Fn1	rs30570886	A	T*	intron_variant _
1	71622906	Fn1	rs30570889	G	T*	intron_variant _
1	71623029	Fn1	rs30570892	A	T*	intron_variant _
1	71623185	Fn1	rs30571601	C	A*	intron_variant _
1	71623541	Fn1	rs221423644	C	T*	intron_variant _
1	71623591	Fn1	rs259996293	G	A*	intron_variant _
1	71623772	Fn1	rs30572507	A	G*	intron_variant _
1	71623868	Fn1	rs30572513	C	A*	intron_variant _
1	71623911	Fn1	rs30573336	C	T*	intron_variant _
1	71624023	Fn1	rs30573339	G	T*	intron_variant _
1	71646396	Fn1	rs30572717	C	A*	intron_variant _
1	71646490	Fn1	rs30572723	C	T*	intron_variant _
1	71646778	Fn1	rs30573606	A	C*	intron_variant _
1	71647014	Fn1	rs30573612	C	G*	intron_variant _
1	71647054	Fn1	rs30574505	T	C*	intron_variant _
1	71647063	Fn1	rs217229455	T	C*	intron_variant _
1	71647151	Fn1	rs234035101	A	G*	intron_variant _
1	71647168	Fn1	rs255400867	A	T*	intron_variant _
1	71647170	Fn1	rs216843715	G	A*	intron_variant _
1	71647307	Fn1	rs30574511	A	G*	intron_variant _
1	71647348	Fn1	rs30574513	G	C*	intron_variant _
1	71647675	Fn1	rs221837436	C	T*	intron_variant _
1	71647747	Fn1	rs6212802	T	C*	intron_variant _
1	71647748	Fn1	rs6212803	C	T*	intron_variant _
1	71647828	Fn1	rs6213280	G	T*	intron_variant _
1	71586345	Fn1	rs243151309	T	G*	intron_variant downstream_gene_variant _
1	71586701	Fn1	rs30563079	T	G*	intron_variant downstream_gene_variant _
1	71586742	Fn1	rs30563082	T	C*	intron_variant downstream_gene_variant _
1	71586953	Fn1	rs30563925	A	G*	intron_variant downstream_gene_variant _
1	71586994	Fn1	rs30563928	T	C*	intron_variant downstream_gene_variant _
1	71587030	Fn1	rs30563931	C	G*	intron_variant downstream_gene_variant _
1	71587067	Fn1	rs30564724	A	G*	intron_variant downstream_gene_variant _
1	71587129	Fn1	rs226726451	G	A*	intron_variant downstream_gene_variant _
1	71587189	Fn1	rs30564730	G	A*	intron_variant downstream_gene_variant _
1	71587348	Fn1	rs214883556	A	G*	intron_variant downstream_gene_variant _
1	71587461	Fn1	rs30564733	C	T*	intron_variant downstream_gene_variant _
1	71587549	Fn1	rs30565516	T	C*	intron_variant downstream_gene_variant _
1	71587965	Fn1	rs30565522	G	A*	intron_variant downstream_gene_variant _
1	71588012	Fn1	rs30566205	G	A*	intron_variant downstream_gene_variant _
1	71588245	Fn1	rs581766791	C	T*	intron_variant downstream_gene_variant _
1	71588247	Fn1	rs579166185	G	C*	intron_variant downstream_gene_variant _
1	71588248	Fn1	rs584140736	G	A*	intron_variant downstream_gene_variant _
1	71588279	Fn1	rs236595418	C	A*	intron_variant downstream_gene_variant _
1	71588281	Fn1	rs248258548	T	C*	intron_variant downstream_gene_variant _
1	71588328	Fn1	rs236711274	G	A*	intron_variant downstream_gene_variant _
1	71588379	Fn1	rs222688697	G	A*	intron_variant downstream_gene_variant _
1	71588425	Fn1	rs235884849	C	T*	intron_variant downstream_gene_variant _
1	71588507	Fn1	rs258836998	A	C*	intron_variant downstream_gene_variant _
1	71588524	Fn1	rs218593079	T	C*	intron_variant downstream_gene_variant _
1	71588735	Fn1	rs230259241	G	A*	intron_variant downstream_gene_variant _

1	71589533	Fn1	rs30564906	C	G*	intron_variant downstream_gene_variant _
1	71589767	Fn1	rs222533050	A	G*	intron_variant downstream_gene_variant _
1	71589809	Fn1	rs249070151	T	c*	intron_variant downstream_gene_variant _
1	71589831	Fn1	rs259159688	T	C*	intron_variant downstream_gene_variant _
1	71589856	Fn1	rs229296541	G	A*	intron_variant downstream_gene_variant _
1	71589861	Fn1	rs242127035	C	G*	intron_variant downstream_gene_variant _
1	71589867	Fn1	rs256955514	A	T*	intron_variant downstream_gene_variant _
1	71589884	Fn1	rs228568554	A	C*	intron_variant downstream_gene_variant _
1	71589900	Fn1	rs251754995	C	T*	intron_variant downstream_gene_variant _
1	71589906	Fn1	rs214567806	C	T*	intron_variant downstream_gene_variant _
1	71589914	Fn1	rs232718153	C	T*	intron_variant downstream_gene_variant _
1	71589915	Fn1	rs245643242	A	G*	intron_variant downstream_gene_variant _
1	71590007	Fn1	rs30564912	A	G*	intron_variant downstream_gene_variant _
1	71590033	Fn1	rs30565845	T	C*	intron_variant downstream_gene_variant _
1	71629939	Fn1	rs30569915	G	A*	intron_variant nmd_transcript_variant
1	71630145	Fn1	-	G	A*	intron_variant nmd_transcript_variant
1	71630166	Fn1	rs31903881	A	G*	intron_variant nmd_transcript_variant
1	71630284	Fn1	rs212660299	C	A*	intron_variant nmd_transcript_variant
1	71630353	Fn1	rs213371501	G	A*	intron_variant nmd_transcript_variant
1	71630354	Fn1	rs234529566	T	C*	intron_variant nmd_transcript_variant
1	71630365	Fn1	rs262756729	G	A*	intron_variant nmd_transcript_variant
1	71630438	Fn1	rs218884950	G	A*	intron_variant nmd_transcript_variant
1	71630443	Fn1	rs31499560	G	A*	intron_variant nmd_transcript_variant
1	71630619	Fn1	rs30570874	G	C*	intron_variant nmd_transcript_variant
1	71630769	Fn1	rs30570877	G	C*	intron_variant nmd_transcript_variant
1	71630989	Fn1	rs233279794	G	C*	intron_variant nmd_transcript_variant
1	71630990	Fn1	rs245345009	G	A*	intron_variant nmd_transcript_variant
1	71631091	Fn1	rs245368268	A	G*	intron_variant nmd_transcript_variant
1	71631169	Fn1	rs216308610	C	T*	intron_variant nmd_transcript_variant
1	71631171	Fn1	rs227784108	A	T*	intron_variant nmd_transcript_variant
1	71631996	Fn1	rs30571775	A	G*	intron_variant nmd_transcript_variant
1	71632301	Fn1	rs231981025	T	C*	intron_variant nmd_transcript_variant
1	71632325	Fn1	rs264250295	A	G*	intron_variant nmd_transcript_variant
1	71632606	Fn1	rs30572754	C	T*	intron_variant nmd_transcript_variant
1	71632616	Fn1	rs30572757	C	T*	intron_variant nmd_transcript_variant
1	71632635	Fn1	rs30572760	T	C*	intron_variant nmd_transcript_variant
1	71632747	Fn1	rs253659667	A	T*	intron_variant nmd_transcript_variant
1	71632760	Fn1	rs32679813	C	T*	intron_variant nmd_transcript_variant
1	71633056	Fn1	rs254662219	A	G*	intron_variant nmd_transcript_variant
1	71633174	Fn1	rs30573646	C	T*	intron_variant nmd_transcript_variant
1	71633708	Fn1	rs31817432	T	C*	intron_variant nmd_transcript_variant
1	71633748	Fn1	rs30574635	T	G*	intron_variant nmd_transcript_variant
1	71633962	Fn1	rs30574640	A	G*	intron_variant nmd_transcript_variant
1	71634051	Fn1	rs225872212	C	T*	intron_variant nmd_transcript_variant
1	71634055	Fn1	rs243635640	C	G*	intron_variant nmd_transcript_variant
1	71634083	Fn1	rs265661068	G	T*	intron_variant nmd_transcript_variant
1	71634101	Fn1	rs30575681	G	T*	intron_variant nmd_transcript_variant
1	71634159	Fn1	rs224990627	C	T*	intron_variant nmd_transcript_variant
1	71634162	Fn1	rs243580750	G	A*	intron_variant nmd_transcript_variant
1	71634194	Fn1	rs30576614	C	T*	intron_variant nmd_transcript_variant
1	71634203	Fn1	rs586794798	G	A*	intron_variant nmd_transcript_variant
1	71634266	Fn1	rs581245652	G	A*	intron_variant nmd_transcript_variant
1	71634389	Fn1	rs259606823	C	T*	intron_variant nmd_transcript_variant
1	71634448	Fn1	rs240552685	T	C*	intron_variant nmd_transcript_variant
1	71634453	Fn1	rs263945641	C	T*	intron_variant nmd_transcript_variant
1	71634477	Fn1	rs30576617	A	G*	intron_variant nmd_transcript_variant
1	71635151	Fn1	rs30576623	T	C*	intron_variant nmd_transcript_variant
1	71635197	Fn1	rs258353177	A	G*	intron_variant nmd_transcript_variant
1	71635528	Fn1	rs260470127	T	C*	intron_variant nmd_transcript_variant
1	71635770	Fn1	rs258267769	T	G*	intron_variant nmd_transcript_variant
1	71635779	Fn1	rs225789748	A	G*	intron_variant nmd_transcript_variant
1	71635952	Fn1	rs230206018	C	A*	intron_variant nmd_transcript_variant
1	71635973	Fn1	rs213990646	A	G*	intron_variant nmd_transcript_variant
1	71635998	Fn1	rs30568797	G	A*	intron_variant nmd_transcript_variant
1	71636029	Fn1	rs30568800	T	C*	intron_variant nmd_transcript_variant
1	71636034	Fn1	rs223729059	T	C*	intron_variant nmd_transcript_variant
1	71636082	Fn1	rs239996949	T	C*	intron_variant nmd_transcript_variant
1	71636125	Fn1	rs218666574	T	G*	intron_variant nmd_transcript_variant
1	71636203	Fn1	rs237068285	G	A*	intron_variant nmd_transcript_variant
1	71636221	Fn1	rs258251596	G	A*	intron_variant nmd_transcript_variant
1	71636231	Fn1	rs219530628	C	T*	intron_variant nmd_transcript_variant
1	71636288	Fn1	rs263175342	A	C*	intron_variant nmd_transcript_variant
1	71636318	Fn1	rs579756432	G	A*	intron_variant nmd_transcript_variant
1	71636322	Fn1	rs235031036	T	C*	intron_variant nmd_transcript_variant
1	71636385	Fn1	rs30568803	G	A*	intron_variant nmd_transcript_variant
1	71636814	Fn1	rs238983206	A	C*	intron_variant nmd_transcript_variant
1	71636863	Fn1	rs32545328	T	G*	intron_variant nmd_transcript_variant
1	71636913	Fn1	rs251885772	G	A*	intron_variant nmd_transcript_variant
1	71636914	Fn1	rs221917576	C	T*	intron_variant nmd_transcript_variant
1	71636941	Fn1	rs246270893	C	T*	intron_variant nmd_transcript_variant
1	71637111	Fn1	rs226680308	A	G*	intron_variant nmd_transcript_variant
1	71637118	Fn1	rs240903862	T	C*	intron_variant nmd_transcript_variant
1	71637145	Fn1	rs254724033	A	G*	intron_variant nmd_transcript_variant

1	71637187	Fn1	rs223627846	C	T*	intron_variant nmd_transcript_variant
1	71637404	Fn1	rs255613188	A	T*	intron_variant nmd_transcript_variant
1	71637551	Fn1	rs233505562	T	A*	intron_variant nmd_transcript_variant
1	71637688	Fn1	rs30569786	C	T*	intron_variant nmd_transcript_variant
1	71590230	Fn1	rs257734139	G	A*	intron_variant nmd_transcript_variant _
1	71590237	Fn1	rs222010016	T	A*	intron_variant nmd_transcript_variant _
1	71590374	Fn1	rs579968228	C	A*	intron_variant nmd_transcript_variant _
1	71590416	Fn1	rs241201811	C	T*	intron_variant nmd_transcript_variant _
1	71590490	Fn1	rs252811446	A	G*	intron_variant nmd_transcript_variant _
1	71590494	Fn1	rs223496534	G	T*	intron_variant nmd_transcript_variant _
1	71590495	Fn1	rs242087870	T	C*	intron_variant nmd_transcript_variant _
1	71590509	Fn1	rs227876441	G	A*	intron_variant nmd_transcript_variant _
1	71590560	Fn1	rs247128705	A	G*	intron_variant nmd_transcript_variant _
1	71590569	Fn1	rs30565853	G	C*	intron_variant nmd_transcript_variant _
1	71590618	Fn1	rs30566856	T	C*	intron_variant nmd_transcript_variant _
1	71590657	Fn1	rs30566858	C	G*	intron_variant nmd_transcript_variant _
1	71590972	Fn1	rs30567790	A	C*	intron_variant nmd_transcript_variant _
1	71637852	Fn1	rs30569789	G	A*	intron_variant nmd_transcript_variant downstream_gene_variant
1	71637862	Fn1	rs30569792	G	A*	intron_variant nmd_transcript_variant downstream_gene_variant
1	71638154	Fn1	rs265106508	C	G*	intron_variant nmd_transcript_variant downstream_gene_variant
1	71638265	Fn1	rs248257356	A	C*	intron_variant nmd_transcript_variant downstream_gene_variant
1	71638422	Fn1	rs30570661	C	T*	intron_variant nmd_transcript_variant downstream_gene_variant
1	71638782	Fn1	rs247598157	G	A*	intron_variant nmd_transcript_variant downstream_gene_variant
1	71638991	Fn1	rs218967607	G	A*	intron_variant nmd_transcript_variant downstream_gene_variant
1	71639089	Fn1	rs265763631	C	G*	intron_variant nmd_transcript_variant downstream_gene_variant
1	71639116	Fn1	rs238444510	A	T*	intron_variant nmd_transcript_variant downstream_gene_variant
1	71639128	Fn1	rs257196773	G	A*	intron_variant nmd_transcript_variant downstream_gene_variant
1	71639162	Fn1	rs245536332	A	G*	intron_variant nmd_transcript_variant downstream_gene_variant
1	71639206	Fn1	rs584148352	A	C*	intron_variant nmd_transcript_variant downstream_gene_variant
1	71639313	Fn1	rs266193016	T	C*	intron_variant nmd_transcript_variant downstream_gene_variant
1	71639552	Fn1	rs30571626	C	T*	intron_variant nmd_transcript_variant downstream_gene_variant
1	71639909	Fn1	rs212033901	A	T*	intron_variant nmd_transcript_variant downstream_gene_variant
1	71639915	Fn1	rs232143734	A	G*	intron_variant nmd_transcript_variant downstream_gene_variant
1	71640144	Fn1	rs253407613	A	G*	intron_variant nmd_transcript_variant downstream_gene_variant
1	71640467	Fn1	rs30572544	C	T*	intron_variant nmd_transcript_variant downstream_gene_variant
1	71640481	Fn1	rs30572546	C	T*	intron_variant nmd_transcript_variant downstream_gene_variant
1	71640563	Fn1	rs264287151	A	C*	intron_variant nmd_transcript_variant downstream_gene_variant
1	71640570	Fn1	rs222846219	T	A*	intron_variant nmd_transcript_variant downstream_gene_variant
1	71640679	Fn1	rs220510775	C	T*	intron_variant nmd_transcript_variant downstream_gene_variant
1	71640700	Fn1	rs30572549	G	A*	intron_variant nmd_transcript_variant downstream_gene_variant
1	71640745	Fn1	rs30572552	G	A*	intron_variant nmd_transcript_variant downstream_gene_variant
1	71640898	Fn1	rs236431238	C	T*	intron_variant nmd_transcript_variant downstream_gene_variant
1	71640900	Fn1	rs253715168	A	C*	intron_variant nmd_transcript_variant downstream_gene_variant
1	71640957	Fn1	rs216644478	T	C*	intron_variant nmd_transcript_variant downstream_gene_variant
1	71641078	Fn1	rs48785794	C	T*	intron_variant nmd_transcript_variant downstream_gene_variant
1	71641079	Fn1	rs236461436	A	G*	intron_variant nmd_transcript_variant downstream_gene_variant
1	71596123	Fn1	rs259596791	C	T*	intron_variant nmd_transcript_variant downstream_gene_variant _
1	71596152	Fn1	rs30568282	T	C*	intron_variant nmd_transcript_variant downstream_gene_variant _
1	71596293	Fn1	rs30569091	T	C*	intron_variant nmd_transcript_variant downstream_gene_variant _
1	71596318	Fn1	rs30569904	A	G*	intron_variant nmd_transcript_variant downstream_gene_variant _
1	71596483	Fn1	rs30569910	T	C*	intron_variant nmd_transcript_variant downstream_gene_variant _
1	71596728	Fn1	rs225843679	G	A*	intron_variant nmd_transcript_variant downstream_gene_variant _
1	71596736	Fn1	rs259316256	C	T*	intron_variant nmd_transcript_variant downstream_gene_variant _
1	71596737	Fn1	rs229407401	A	T*	intron_variant nmd_transcript_variant downstream_gene_variant _
1	71596787	Fn1	rs213632419	C	A*	intron_variant nmd_transcript_variant downstream_gene_variant _
1	71596897	Fn1	rs212280271	A	G*	intron_variant nmd_transcript_variant downstream_gene_variant _
1	71596969	Fn1	rs234606363	A	T*	intron_variant nmd_transcript_variant downstream_gene_variant _
1	71596970	Fn1	rs262442814	C	T*	intron_variant nmd_transcript_variant downstream_gene_variant _
1	71596971	Fn1	rs219607115	T	C*	intron_variant nmd_transcript_variant downstream_gene_variant _
1	71597023	Fn1	rs587164306	G	A*	intron_variant nmd_transcript_variant downstream_gene_variant _
1	71597036	Fn1	rs223610436	A	G*	intron_variant nmd_transcript_variant downstream_gene_variant _
1	71597075	Fn1	rs251837712	A	G*	intron_variant nmd_transcript_variant downstream_gene_variant _
1	71597078	Fn1	rs255839194	G	C*	intron_variant nmd_transcript_variant downstream_gene_variant _
1	71597857	Fn1	rs225359437	A	C*	intron_variant nmd_transcript_variant downstream_gene_variant _
1	71598137	Fn1	rs264910548	T	C*	intron_variant nmd_transcript_variant downstream_gene_variant _
1	71598263	Fn1	rs30571889	A	T*	intron_variant nmd_transcript_variant downstream_gene_variant _
1	71598286	Fn1	rs30564485	G	A*	intron_variant nmd_transcript_variant downstream_gene_variant _
1	71598490	Fn1	rs234605701	C	T*	intron_variant nmd_transcript_variant downstream_gene_variant _
1	71598603	Fn1	rs30564491	A	C*	intron_variant nmd_transcript_variant downstream_gene_variant _
1	71598633	Fn1	rs30565234	T	C*	intron_variant nmd_transcript_variant downstream_gene_variant _
1	71598859	Fn1	rs252811355	T	C*	intron_variant nmd_transcript_variant downstream_gene_variant _
1	71598904	Fn1	rs30565243	T	G*	intron_variant nmd_transcript_variant downstream_gene_variant _
1	71598951	Fn1	rs30566146	C	T*	intron_variant nmd_transcript_variant downstream_gene_variant _
1	71599054	Fn1	rs220303661	T	C*	intron_variant nmd_transcript_variant downstream_gene_variant _
1	71599056	Fn1	rs249974815	C	T*	intron_variant nmd_transcript_variant downstream_gene_variant _
1	71599062	Fn1	rs221624701	T	C*	intron_variant nmd_transcript_variant downstream_gene_variant _
1	71599082	Fn1	rs239560253	C	T*	intron_variant nmd_transcript_variant downstream_gene_variant _
1	71599434	Fn1	rs30566895	T	G*	intron_variant nmd_transcript_variant downstream_gene_variant _
1	71599486	Fn1	rs227854437	T	A*	intron_variant nmd_transcript_variant downstream_gene_variant _
1	71599514	Fn1	rs580935650	A	G*	intron_variant nmd_transcript_variant downstream_gene_variant _
1	71599542	Fn1	rs584679923	C	G*	intron_variant nmd_transcript_variant downstream_gene_variant _
1	71599578	Fn1	rs30566898	T	C*	intron_variant nmd_transcript_variant downstream_gene_variant _
1	71599749	Fn1	rs30566900	A	G*	intron_variant nmd_transcript_variant downstream_gene_variant _

Disease Models & Mechanisms • Supplementary information

1	71599790	Fn1	rs239502075	C	T*	intron_variant nmd_transcript_variant downstream_gene_variant _
1	71599849	Fn1	rs30566903	T	A*	intron_variant nmd_transcript_variant downstream_gene_variant _
1	71599938	Fn1	rs30568845	C	T*	intron_variant nmd_transcript_variant downstream_gene_variant _
1	71600122	Fn1	rs242682071	T	C*	intron_variant nmd_transcript_variant downstream_gene_variant _
1	71600197	Fn1	rs264764663	G	A*	intron_variant nmd_transcript_variant downstream_gene_variant _
1	71600204	Fn1	rs30568847	T	C*	intron_variant nmd_transcript_variant downstream_gene_variant _
1	71591130	Fn1	rs30568646	T	C*	intron_variant nmd_transcript_variant upstream_gene_variant _
1	71591138	Fn1	rs220067544	A	T*	intron_variant nmd_transcript_variant upstream_gene_variant _
1	71591175	Fn1	rs223423433	T	A*	intron_variant nmd_transcript_variant upstream_gene_variant _
1	71591191	Fn1	rs235544700	G	A*	intron_variant nmd_transcript_variant upstream_gene_variant _
1	71591334	Fn1	rs244277382	T	C*	intron_variant nmd_transcript_variant upstream_gene_variant _
1	71591341	Fn1	rs265121097	T	G*	intron_variant nmd_transcript_variant upstream_gene_variant _
1	71591455	Fn1	rs30568649	G	A*	intron_variant nmd_transcript_variant upstream_gene_variant _
1	71591489	Fn1	rs30568652	A	T*	intron_variant nmd_transcript_variant upstream_gene_variant _
1	71591527	Fn1	rs586595202	C	A*	intron_variant nmd_transcript_variant upstream_gene_variant _
1	71591528	Fn1	rs581221208	A	G*	intron_variant nmd_transcript_variant upstream_gene_variant _
1	71591870	Fn1	rs30569548	T	C*	intron_variant nmd_transcript_variant upstream_gene_variant _
1	71592139	Fn1	rs30570444	A	C*	intron_variant nmd_transcript_variant upstream_gene_variant _
1	71592406	Fn1	rs260145196	T	A*	intron_variant nmd_transcript_variant upstream_gene_variant _
1	71592500	Fn1	rs264754590	C	T*	intron_variant nmd_transcript_variant upstream_gene_variant _
1	71592802	Fn1	rs30570447	A	G*	intron_variant nmd_transcript_variant upstream_gene_variant _
1	71593320	Fn1	rs30571377	T	C*	intron_variant nmd_transcript_variant upstream_gene_variant _
1	71593476	Fn1	rs30571383	C	T*	intron_variant nmd_transcript_variant upstream_gene_variant downstream_gene_variant _
1	71593501	Fn1	rs30572256	C	T*	intron_variant nmd_transcript_variant upstream_gene_variant downstream_gene_variant _
1	71593622	Fn1	rs30572259	G	A*	intron_variant nmd_transcript_variant upstream_gene_variant downstream_gene_variant _
1	71593640	Fn1	rs30564076	A	C*	intron_variant nmd_transcript_variant upstream_gene_variant downstream_gene_variant _
1	71593975	Fn1	rs262979969	A	G*	intron_variant nmd_transcript_variant upstream_gene_variant downstream_gene_variant _
1	71594020	Fn1	rs582402279	A	G*	intron_variant nmd_transcript_variant upstream_gene_variant downstream_gene_variant _
1	71594340	Fn1	rs30564855	A	C*	intron_variant nmd_transcript_variant upstream_gene_variant downstream_gene_variant _
1	71594555	Fn1	rs30564860	A	T*	intron_variant nmd_transcript_variant upstream_gene_variant downstream_gene_variant _
1	71594741	Fn1	rs30565609	T	C*	intron_variant nmd_transcript_variant upstream_gene_variant downstream_gene_variant _
1	71594946	Fn1	rs30566505	A	G*	intron_variant nmd_transcript_variant upstream_gene_variant downstream_gene_variant _
1	71595074	Fn1	rs258097166	A	C*	intron_variant nmd_transcript_variant upstream_gene_variant downstream_gene_variant _
1	71595130	Fn1	rs219411018	G	A*	intron_variant nmd_transcript_variant upstream_gene_variant downstream_gene_variant _
1	71595144	Fn1	rs226812504	T	C*	intron_variant nmd_transcript_variant upstream_gene_variant downstream_gene_variant _
1	71595165	Fn1	rs247460174	T	C*	intron_variant nmd_transcript_variant upstream_gene_variant downstream_gene_variant _
1	71595840	Fn1	rs30568276	G	A*	intron_variant nmd_transcript_variant upstream_gene_variant downstream_gene_variant _
1	71595982	Fn1	rs241255915	T	C*	intron_variant nmd_transcript_variant upstream_gene_variant downstream_gene_variant _
1	71596058	Fn1	rs225945078	G	A*	intron_variant nmd_transcript_variant upstream_gene_variant downstream_gene_variant _
1	71596060	Fn1	rs233559942	T	C*	intron_variant nmd_transcript_variant upstream_gene_variant downstream_gene_variant _
1	71596061	Fn1	rs259198754	G	A*	intron_variant nmd_transcript_variant upstream_gene_variant downstream_gene_variant _
1	71600816	Fn1	rs233045403	G	A*	intron_variant nmd_transcript_variant upstream_gene_variant downstream_gene_variant _
1	71600939	Fn1	rs30569942	T	C*	intron_variant nmd_transcript_variant upstream_gene_variant downstream_gene_variant _
1	71600980	Fn1	rs217840222	C	G*	intron_variant nmd_transcript_variant upstream_gene_variant downstream_gene_variant _
1	71601052	Fn1	rs257263902	T	C*	intron_variant nmd_transcript_variant upstream_gene_variant downstream_gene_variant _
1	71601056	Fn1	rs221575445	C	T*	intron_variant nmd_transcript_variant upstream_gene_variant downstream_gene_variant _
1	71601061	Fn1	rs244985236	T	C*	intron_variant nmd_transcript_variant upstream_gene_variant downstream_gene_variant _
1	71601083	Fn1	rs30570829	G	A*	intron_variant nmd_transcript_variant upstream_gene_variant downstream_gene_variant _
1	71601303	Fn1	rs216038927	A	C*	intron_variant nmd_transcript_variant upstream_gene_variant downstream_gene_variant _
1	71601337	Fn1	rs30571618	C	T*	intron_variant nmd_transcript_variant upstream_gene_variant downstream_gene_variant _
1	71601449	Fn1	rs30571621	G	T*	intron_variant nmd_transcript_variant upstream_gene_variant downstream_gene_variant _
1	71601521	Fn1	rs261135837	G	T*	intron_variant nmd_transcript_variant upstream_gene_variant downstream_gene_variant _
1	71601585	Fn1	rs232322194	A	C*	intron_variant nmd_transcript_variant upstream_gene_variant downstream_gene_variant _
1	71601619	Fn1	rs221491406	G	A*	intron_variant nmd_transcript_variant upstream_gene_variant downstream_gene_variant _
1	71601664	Fn1	rs30572514	T	C*	intron_variant nmd_transcript_variant upstream_gene_variant downstream_gene_variant _
1	71601737	Fn1	rs30572517	A	G*	intron_variant nmd_transcript_variant upstream_gene_variant downstream_gene_variant _
1	71601771	Fn1	rs253822699	G	A*	intron_variant nmd_transcript_variant upstream_gene_variant downstream_gene_variant _
1	71601842	Fn1	rs243806467	G	A*	intron_variant nmd_transcript_variant upstream_gene_variant downstream_gene_variant _
1	71601949	Fn1	rs258135940	C	T*	intron_variant nmd_transcript_variant upstream_gene_variant downstream_gene_variant _
1	71602082	Fn1	rs260354662	T	G*	intron_variant nmd_transcript_variant upstream_gene_variant downstream_gene_variant _
1	71602096	Fn1	rs218246726	G	A*	intron_variant nmd_transcript_variant upstream_gene_variant downstream_gene_variant _
1	71602245	Fn1	rs232194031	T	C*	intron_variant nmd_transcript_variant upstream_gene_variant downstream_gene_variant _
1	71602299	Fn1	rs253960386	T	G*	intron_variant nmd_transcript_variant upstream_gene_variant downstream_gene_variant _
1	71624425	Fn1	rs30574141	G	A*	intron_variant upstream_gene_variant
1	71604437	Fn1	rs580116367	C	T*	intron_variant upstream_gene_variant _
1	71604442	Fn1	rs583342827	G	A*	intron_variant upstream_gene_variant _
1	71604459	Fn1	rs586404270	T	C*	intron_variant upstream_gene_variant _
1	71604460	Fn1	rs580429469	A	T*	intron_variant upstream_gene_variant _
1	71604462	Fn1	rs583217278	C	A*	intron_variant upstream_gene_variant _
1	71604495	Fn1	rs586589034	G	A*	intron_variant upstream_gene_variant _
1	71604500	Fn1	rs230439278	C	T*	intron_variant upstream_gene_variant _
1	71604575	Fn1	rs214165557	G	A*	intron_variant upstream_gene_variant _
1	71604661	Fn1	rs30568503	C	A*	intron_variant upstream_gene_variant _
1	71604739	Fn1	rs30569456	A	G*	intron_variant upstream_gene_variant _
1	71604801	Fn1	rs240247427	C	T*	intron_variant upstream_gene_variant _
1	71604822	Fn1	rs263742276	A	T*	intron_variant upstream_gene_variant _
1	71604853	Fn1	rs237247987	G	A*	intron_variant upstream_gene_variant _
1	71604863	Fn1	rs30569462	T	C*	intron_variant upstream_gene_variant _
1	71604920	Fn1	rs30570295	C	T*	intron_variant upstream_gene_variant _
1	71604947	Fn1	rs30570298	G	A*	intron_variant upstream_gene_variant _
1	71605128	Fn1	rs214130803	C	T*	intron_variant upstream_gene_variant _
1	71605416	Fn1	rs586947177	C	T*	intron_variant upstream_gene_variant _
1	71605418	Fn1	rs219639303	C	T*	intron_variant upstream_gene_variant _

Disease Models & Mechanisms • Supplementary information

1	71605431	Fn1	rs241392496	C	T*	intron_variant upstream_gene_variant _
1	71605442	Fn1	rs265555369	G	C*	intron_variant upstream_gene_variant _
1	71605647	Fn1	rs30571203	T	C*	intron_variant upstream_gene_variant _
1	71605736	Fn1	rs30572059	C	T*	intron_variant upstream_gene_variant _
1	71605800	Fn1	rs255881351	C	T*	intron_variant upstream_gene_variant _
1	71605871	Fn1	rs30572828	G	C*	intron_variant upstream_gene_variant _
1	71606082	Fn1	rs252043949	G	C*	intron_variant upstream_gene_variant _
1	71606129	Fn1	rs30564853	T	A*	intron_variant upstream_gene_variant _
1	71606133	Fn1	rs241134477	C	T*	intron_variant upstream_gene_variant _
1	71606250	Fn1	rs218210684	A	G*	intron_variant upstream_gene_variant _
1	71606279	Fn1	rs30566036	T	C*	intron_variant upstream_gene_variant _
1	71606346	Fn1	rs30566042	G	C*	intron_variant upstream_gene_variant _
1	71606389	Fn1	rs213606433	C	T*	intron_variant upstream_gene_variant _
1	71606392	Fn1	rs238695054	A	G*	intron_variant upstream_gene_variant _
1	71606393	Fn1	rs262939770	C	T*	intron_variant upstream_gene_variant _
1	71606413	Fn1	rs219299744	G	A*	intron_variant upstream_gene_variant _
1	71606517	Fn1	rs578386890	T	G*	intron_variant upstream_gene_variant _
1	71606622	Fn1	rs30567008	T	C*	intron_variant upstream_gene_variant _
1	71606652	Fn1	rs30567011	G	A*	intron_variant upstream_gene_variant _
1	71606883	Fn1	rs30567907	T	C*	intron_variant upstream_gene_variant _
1	71606970	Fn1	rs30567913	A	G*	intron_variant upstream_gene_variant _
1	71607575	Fn1	rs225242803	A	G*	intron_variant upstream_gene_variant _
1	71607601	Fn1	rs240394766	T	G*	intron_variant upstream_gene_variant _
1	71607616	Fn1	rs261191978	T	A*	intron_variant upstream_gene_variant _
1	71607629	Fn1	rs226677113	A	T*	intron_variant upstream_gene_variant _
1	71607705	Fn1	rs582618525	A	g*	intron_variant upstream_gene_variant _
1	71607791	Fn1	rs580614591	G	T*	intron_variant upstream_gene_variant _
1	71607792	Fn1	rs583988197	G	T*	intron_variant upstream_gene_variant _
1	71609748	Fn1	-	G	C*	intron_variant upstream_gene_variant _
1	71609770	Fn1	rs30571102	A	C*	intron_variant upstream_gene_variant _
1	71609909	Fn1	rs30572005	G	A*	intron_variant upstream_gene_variant _
1	71609963	Fn1	rs214745431	T	C*	intron_variant upstream_gene_variant _
1	71609981	Fn1	rs232322244	C	T*	intron_variant upstream_gene_variant _
1	71609985	Fn1	rs256764259	A	C*	intron_variant upstream_gene_variant _
1	71610063	Fn1	rs264101705	T	A*	intron_variant upstream_gene_variant _
1	71610239	Fn1	rs263591475	T	C*	intron_variant upstream_gene_variant _
1	71610271	Fn1	rs224307249	T	C*	intron_variant upstream_gene_variant _
1	71610295	Fn1	rs248490519	T	G*	intron_variant upstream_gene_variant _
1	71610517	Fn1	rs30572008	T	C*	intron_variant upstream_gene_variant _
1	71610549	Fn1	rs30572010	A	C*	intron_variant upstream_gene_variant _
1	71610574	Fn1	rs30572013	G	A*	intron_variant upstream_gene_variant _
1	71610638	Fn1	rs587297060	G	A*	intron_variant upstream_gene_variant _
1	71610647	Fn1	rs581774724	C	A*	intron_variant upstream_gene_variant _
1	71610751	Fn1	rs30572986	G	C*	intron_variant upstream_gene_variant _
1	71610830	Fn1	rs248017440	A	C*	intron_variant upstream_gene_variant _
1	71610834	Fn1	rs219959941	T	A*	intron_variant upstream_gene_variant _
1	71611106	Fn1	rs30567976	C	T*	intron_variant upstream_gene_variant _
1	71611152	Fn1	rs30567979	G	C*	intron_variant upstream_gene_variant _
1	71611156	Fn1	rs234263685	G	A*	intron_variant upstream_gene_variant _
1	71611161	Fn1	rs247125399	G	A*	intron_variant upstream_gene_variant _
1	71611184	Fn1	rs30567982	G	A*	intron_variant upstream_gene_variant _
1	71611476	Fn1	rs30568918	C	A*	intron_variant upstream_gene_variant _
1	71611650	Fn1	rs30569757	T	C*	intron_variant upstream_gene_variant _
1	71611677	Fn1	-	C	T*	intron_variant upstream_gene_variant _
1	71611708	Fn1	rs30569759	T	C*	intron_variant upstream_gene_variant _
1	71611765	Fn1	rs235671108	G	A*	intron_variant upstream_gene_variant _
1	71611984	Fn1	rs583006661	C	T*	intron_variant upstream_gene_variant _
1	71612086	Fn1	rs30570778	C	T*	intron_variant upstream_gene_variant _
1	71612101	Fn1	rs30570781	A	G*	intron_variant upstream_gene_variant _
1	71612268	Fn1	rs30571544	G	A*	intron_variant upstream_gene_variant _
1	71613055	Fn1	rs30571553	A	C*	intron_variant upstream_gene_variant _
1	71613662	Fn1	rs30573327	C	T*	intron_variant upstream_gene_variant _
1	71614169	Fn1	rs229058688	G	A*	intron_variant upstream_gene_variant _
1	71614171	Fn1	rs247871528	A	G*	intron_variant upstream_gene_variant _
1	71614227	Fn1	rs30573333	A	C*	intron_variant upstream_gene_variant _
1	71645806	Fn1	rs30570177	G	C*	intron_variant upstream_gene_variant _
1	71645927	Fn1	rs30571019	A	G*	intron_variant upstream_gene_variant _
1	71646128	Fn1	rs30572714	A	G*	intron_variant upstream_gene_variant _
1	71647869	Fn1	rs30576808	C	G*	intron_variant upstream_gene_variant _
1	71647983	Fn1	rs30576811	T	C*	intron_variant upstream_gene_variant _
1	71648396	Fn1	rs30571404	T	A*	intron_variant upstream_gene_variant _
1	71648436	Fn1	rs30571407	G	T*	intron_variant upstream_gene_variant _
1	71648459	Fn1	rs30571410	C	T*	intron_variant upstream_gene_variant _
1	71648625	Fn1	rs30572296	G	A*	intron_variant upstream_gene_variant _
1	71648710	Fn1	rs30572299	G	A*	intron_variant upstream_gene_variant _
1	71648750	Fn1	rs30573164	A	G*	intron_variant upstream_gene_variant _
1	71648788	Fn1	rs260717761	C	T*	intron_variant upstream_gene_variant _
1	71648831	Fn1	rs30573167	G	A*	intron_variant upstream_gene_variant _
1	71648887	Fn1	rs30573170	C	T*	intron_variant upstream_gene_variant _
1	71649020	Fn1	rs253525092	C	T*	intron_variant upstream_gene_variant _
1	71649033	Fn1	rs30574908	C	T*	intron_variant upstream_gene_variant _
1	71649171	Fn1	rs215422462	G	A*	intron_variant upstream_gene_variant _
1	71649177	Fn1	rs229492067	A	C*	intron_variant upstream_gene_variant _

Disease Models & Mechanisms • Supplementary information

1	71649182	Fn1	rs213228582	C	G*	intron_variant upstream_gene_variant _
1	71649489	Fn1	rs254374306	A	G*	intron_variant upstream_gene_variant _
1	71649847	Fn1	rs30577535	G	A*	intron_variant upstream_gene_variant _
1	71650358	Fn1	-	T	G*	intron_variant upstream_gene_variant _
1	71650374	Fn1	rs585266378	G	A*	intron_variant upstream_gene_variant _
1	71650426	Fn1	rs30578326	G	A*	intron_variant upstream_gene_variant _
1	71650456	Fn1	rs30578329	T	C*	intron_variant upstream_gene_variant _
1	71650470	Fn1	rs30578332	T	C*	intron_variant upstream_gene_variant _
1	71650493	Fn1	rs219407290	A	C*	intron_variant upstream_gene_variant _
1	71650499	Fn1	rs240112307	A	G*	intron_variant upstream_gene_variant _
1	71650535	Fn1	rs254503218	A	C*	intron_variant upstream_gene_variant _
1	71650538	Fn1	rs30579235	A	C*	intron_variant upstream_gene_variant _
1	71651210	Fn1	rs30571422	T	C*	intron_variant upstream_gene_variant _
1	71651360	Fn1	rs30572358	T	C*	intron_variant upstream_gene_variant _
1	71651759	Fn1	rs231537327	A	C*	intron_variant upstream_gene_variant _
1	71651824	Fn1	rs30573184	A	G*	intron_variant upstream_gene_variant _
1	71651840	Fn1	rs30573187	A	G*	intron_variant upstream_gene_variant _
1	71651910	Fn1	rs30573193	T	A*	intron_variant upstream_gene_variant _
1	71652022	Fn1	rs30574096	C	A*	intron_variant upstream_gene_variant _
1	71652071	Fn1	rs244438686	C	T*	intron_variant upstream_gene_variant _
1	71652241	Fn1	rs583834233	G	T*	intron_variant upstream_gene_variant _
1	71652326	Fn1	rs30575005	C	G*	intron_variant upstream_gene_variant _
1	71652544	Fn1	rs30575008	T	C*	intron_variant upstream_gene_variant _
1	71652646	Fn1	rs30575011	A	C*	intron_variant upstream_gene_variant _
1	71652699	Fn1	rs30575854	G	A*	intron_variant upstream_gene_variant _
1	71652812	Fn1	rs30575857	A	G*	intron_variant upstream_gene_variant _
1	71624484	Fn1	rs221414473	C	T*	intron_variant upstream_gene_variant downstream_gene_variant
1	71624486	Fn1	rs239244906	T	G*	intron_variant upstream_gene_variant downstream_gene_variant
1	71624488	Fn1	rs259928337	G	A*	intron_variant upstream_gene_variant downstream_gene_variant
1	71624504	Fn1	rs226298720	A	G*	intron_variant upstream_gene_variant downstream_gene_variant
1	71624652	Fn1	rs264688246	A	G*	intron_variant upstream_gene_variant downstream_gene_variant
1	71624725	Fn1	rs231082285	G	A*	intron_variant upstream_gene_variant downstream_gene_variant
1	71624787	Fn1	rs30575050	T	C*	intron_variant upstream_gene_variant downstream_gene_variant
1	71625108	Fn1	rs236815494	G	A*	intron_variant upstream_gene_variant downstream_gene_variant
1	71625122	Fn1	rs30575946	A	G*	intron_variant upstream_gene_variant downstream_gene_variant
1	71625142	Fn1	rs30575949	A	C*	intron_variant upstream_gene_variant downstream_gene_variant
1	71625185	Fn1	rs253133583	C	T*	intron_variant upstream_gene_variant downstream_gene_variant
1	71625189	Fn1	rs225744086	G	A*	intron_variant upstream_gene_variant downstream_gene_variant
1	71625241	Fn1	rs30575952	T	C*	intron_variant upstream_gene_variant downstream_gene_variant
1	71625249	Fn1	rs255566530	T	G*	intron_variant upstream_gene_variant downstream_gene_variant
1	71625340	Fn1	rs30568988	T	C*	intron_variant upstream_gene_variant downstream_gene_variant
1	71625562	Fn1	rs586777495	G	A*	intron_variant upstream_gene_variant downstream_gene_variant
1	71625603	Fn1	rs30569984	T	A*	intron_variant upstream_gene_variant downstream_gene_variant
1	71625626	Fn1	-	A	G*	intron_variant upstream_gene_variant downstream_gene_variant
1	71625634	Fn1	rs243037589	G	A*	intron_variant upstream_gene_variant downstream_gene_variant
1	71625712	Fn1	rs30569990	C	T*	intron_variant upstream_gene_variant downstream_gene_variant
1	71625836	Fn1	rs30569993	T	C*	intron_variant upstream_gene_variant downstream_gene_variant
1	71625855	Fn1	rs30570699	A	T*	intron_variant upstream_gene_variant downstream_gene_variant
1	71625904	Fn1	rs30570702	C	A*	intron_variant upstream_gene_variant downstream_gene_variant
1	71625922	Fn1	rs223550404	T	G*	intron_variant upstream_gene_variant downstream_gene_variant
1	71625930	Fn1	rs238995631	G	A*	intron_variant upstream_gene_variant downstream_gene_variant
1	71626262	Fn1	rs30571468	G	A*	intron_variant upstream_gene_variant downstream_gene_variant
1	71626554	Fn1	rs30571471	G	A*	intron_variant upstream_gene_variant downstream_gene_variant
1	71626652	Fn1	rs30572344	C	A*	intron_variant upstream_gene_variant downstream_gene_variant
1	71626760	Fn1	rs237217203	G	A*	intron_variant upstream_gene_variant downstream_gene_variant
1	71626823	Fn1	rs214392216	C	T*	intron_variant upstream_gene_variant downstream_gene_variant
1	71626868	Fn1	rs253682053	G	A*	intron_variant upstream_gene_variant downstream_gene_variant
1	71626875	Fn1	rs236611832	T	C*	intron_variant upstream_gene_variant downstream_gene_variant
1	71626943	Fn1	rs584935326	C	T*	intron_variant upstream_gene_variant downstream_gene_variant
1	71626987	Fn1	rs244390379	A	T*	intron_variant upstream_gene_variant downstream_gene_variant
1	71627033	Fn1	rs237128131	T	C*	intron_variant upstream_gene_variant downstream_gene_variant
1	71627075	Fn1	rs30572349	G	A*	intron_variant upstream_gene_variant downstream_gene_variant
1	71627674	Fn1	rs30573198	T	C*	intron_variant upstream_gene_variant downstream_gene_variant
1	71627695	Fn1	rs221309418	T	C*	intron_variant upstream_gene_variant downstream_gene_variant
1	71627713	Fn1	rs30573201	G	A*	intron_variant upstream_gene_variant downstream_gene_variant
1	71627743	Fn1	rs30574174	A	G*	intron_variant upstream_gene_variant downstream_gene_variant
1	71627824	Fn1	rs241009612	A	G*	intron_variant upstream_gene_variant downstream_gene_variant
1	71627830	Fn1	rs258255076	G	A*	intron_variant upstream_gene_variant downstream_gene_variant
1	71627974	Fn1	rs259265944	A	G*	intron_variant upstream_gene_variant downstream_gene_variant
1	71627996	Fn1	rs212955852	T	G*	intron_variant upstream_gene_variant downstream_gene_variant
1	71627997	Fn1	rs236525342	G	A*	intron_variant upstream_gene_variant downstream_gene_variant
1	71627998	Fn1	rs30574180	A	G*	intron_variant upstream_gene_variant downstream_gene_variant
1	71628220	Fn1	rs579175984	C	T*	intron_variant upstream_gene_variant downstream_gene_variant
1	71628225	Fn1	rs581999902	A	T*	intron_variant upstream_gene_variant downstream_gene_variant
1	71628252	Fn1	rs30574183	C	A*	intron_variant upstream_gene_variant downstream_gene_variant
1	71628299	Fn1	rs30575016	T	C*	intron_variant upstream_gene_variant downstream_gene_variant
1	71628301	Fn1	rs248006922	T	C*	intron_variant upstream_gene_variant downstream_gene_variant
1	71628609	Fn1	rs30575022	A	G*	intron_variant upstream_gene_variant downstream_gene_variant
1	71628682	Fn1	rs213395899	T	G*	intron_variant upstream_gene_variant downstream_gene_variant
1	71628692	Fn1	rs227871769	C	T*	intron_variant upstream_gene_variant downstream_gene_variant
1	71628710	Fn1	rs213829500	T	C*	intron_variant upstream_gene_variant downstream_gene_variant
1	71628724	Fn1	rs30575965	A	C*	intron_variant upstream_gene_variant downstream_gene_variant
1	71628978	Fn1	rs242596432	C	T*	intron_variant upstream_gene_variant downstream_gene_variant

Disease Models & Mechanisms • Supplementary information

1	71629026	Fn1	rs259927194	G	A*	intron_variant upstream_gene_variant downstream_gene_variant
1	71629087	Fn1	rs30575968	T	C*	intron_variant upstream_gene_variant downstream_gene_variant
1	71629110	Fn1	rs219377994	C	A*	intron_variant upstream_gene_variant downstream_gene_variant
1	71629249	Fn1	rs30576844	T	C*	intron_variant upstream_gene_variant downstream_gene_variant
1	71641429	Fn1	rs30573505	T	C*	intron_variant upstream_gene_variant downstream_gene_variant
1	71641704	Fn1	rs30573508	C	T*	intron_variant upstream_gene_variant downstream_gene_variant
1	71641824	Fn1	rs30574387	T	C*	intron_variant upstream_gene_variant downstream_gene_variant
1	71642140	Fn1	rs30575202	A	G*	intron_variant upstream_gene_variant downstream_gene_variant
1	71642151	Fn1	rs30576174	T	C*	intron_variant upstream_gene_variant downstream_gene_variant
1	71642225	Fn1	rs30576177	T	C*	intron_variant upstream_gene_variant downstream_gene_variant
1	71642379	Fn1	rs30568765	C	T*	intron_variant upstream_gene_variant downstream_gene_variant
1	71642485	Fn1	rs222705340	A	G*	intron_variant upstream_gene_variant downstream_gene_variant
1	71642602	Fn1	rs30568770	G	A*	intron_variant upstream_gene_variant downstream_gene_variant
1	71642617	Fn1	rs30568773	T	C*	intron_variant upstream_gene_variant downstream_gene_variant
1	71642666	Fn1	rs30569809	T	G*	intron_variant upstream_gene_variant downstream_gene_variant
1	71642713	Fn1	rs231840134	T	C*	intron_variant upstream_gene_variant downstream_gene_variant
1	71642777	Fn1	rs586390332	C	T*	intron_variant upstream_gene_variant downstream_gene_variant
1	71642811	Fn1	rs256681324	C	T*	intron_variant upstream_gene_variant downstream_gene_variant
1	71643262	Fn1	rs222590765	C	T*	intron_variant upstream_gene_variant downstream_gene_variant
1	71643263	Fn1	rs249185827	A	G*	intron_variant upstream_gene_variant downstream_gene_variant
1	71643355	Fn1	rs30571327	T	C*	intron_variant upstream_gene_variant downstream_gene_variant
1	71643445	Fn1	rs254788532	C	T*	intron_variant upstream_gene_variant downstream_gene_variant
1	71643761	Fn1	rs30572192	G	C*	intron_variant upstream_gene_variant downstream_gene_variant
1	71643762	Fn1	rs236077991	C	T*	intron_variant upstream_gene_variant downstream_gene_variant
1	71643901	Fn1	rs246428303	C	T*	intron_variant upstream_gene_variant downstream_gene_variant
1	71643980	Fn1	rs30573904	C	T*	intron_variant upstream_gene_variant downstream_gene_variant
1	71644014	Fn1	rs30573910	A	G*	intron_variant upstream_gene_variant downstream_gene_variant
1	71644029	Fn1	rs233470900	C	T*	intron_variant upstream_gene_variant downstream_gene_variant
1	71644049	Fn1	rs30573913	G	A*	intron_variant upstream_gene_variant downstream_gene_variant
1	71644238	Fn1	rs30574782	A	G*	intron_variant upstream_gene_variant downstream_gene_variant
1	71644341	Fn1	rs30575684	C	A*	intron_variant upstream_gene_variant downstream_gene_variant
1	71644351	Fn1	rs222664521	G	A*	intron_variant upstream_gene_variant downstream_gene_variant
1	71644391	Fn1	rs30575686	G	A*	intron_variant upstream_gene_variant downstream_gene_variant
1	71644406	Fn1	rs30575689	G	A*	intron_variant upstream_gene_variant downstream_gene_variant
1	71644449	Fn1	rs30575692	A	g*	intron_variant upstream_gene_variant downstream_gene_variant
1	71602567	Fn1	rs30564865	G	T*	intron_variant upstream_gene_variant downstream_gene_variant _
1	71602641	Fn1	rs30564870	G	A*	intron_variant upstream_gene_variant downstream_gene_variant _
1	71602642	Fn1	rs248017301	A	G*	intron_variant upstream_gene_variant downstream_gene_variant _
1	71602807	Fn1	-	C	T*	intron_variant upstream_gene_variant downstream_gene_variant _
1	71602979	Fn1	rs30566784	T	C*	intron_variant upstream_gene_variant downstream_gene_variant _
1	71603098	Fn1	rs229011305	A	G*	intron_variant upstream_gene_variant downstream_gene_variant _
1	71603143	Fn1	rs30566793	A	G*	intron_variant upstream_gene_variant downstream_gene_variant _
1	71603155	Fn1	rs30567666	A	G*	intron_variant upstream_gene_variant downstream_gene_variant _
1	71603213	Fn1	rs247588247	T	C*	intron_variant upstream_gene_variant downstream_gene_variant _
1	71603216	Fn1	rs243542071	A	G*	intron_variant upstream_gene_variant downstream_gene_variant _
1	71603237	Fn1	rs30567669	A	G*	intron_variant upstream_gene_variant downstream_gene_variant _
1	71603310	Fn1	rs217590858	A	C*	intron_variant upstream_gene_variant downstream_gene_variant _
1	71603383	Fn1	rs252412465	C	T*	intron_variant upstream_gene_variant downstream_gene_variant _
1	71603426	Fn1	rs251335377	G	A*	intron_variant upstream_gene_variant downstream_gene_variant _
1	71603472	Fn1	rs585473374	A	G*	intron_variant upstream_gene_variant downstream_gene_variant _
1	71603497	Fn1	rs582141050	A	T*	intron_variant upstream_gene_variant downstream_gene_variant _
1	71603508	Fn1	rs229333576	A	G*	intron_variant upstream_gene_variant downstream_gene_variant _
1	71603521	Fn1	rs258526723	T	G*	intron_variant upstream_gene_variant downstream_gene_variant _
1	71603528	Fn1	rs225983787	A	C*	intron_variant upstream_gene_variant downstream_gene_variant _
1	71603541	Fn1	rs247498077	A	G*	intron_variant upstream_gene_variant downstream_gene_variant _
1	71604023	Fn1	rs30568498	C	A*	intron_variant upstream_gene_variant downstream_gene_variant _
1	71644830	Fn1	rs30576415	G	A*	intron_variant upstream_gene_variant downstream_gene_variant _
1	71645319	Fn1	rs248915366	T	C*	intron_variant upstream_gene_variant downstream_gene_variant _
1	71645343	Fn1	rs239084213	A	G*	intron_variant upstream_gene_variant downstream_gene_variant _
1	71645430	Fn1	rs30576418	C	T*	intron_variant upstream_gene_variant downstream_gene_variant _
1	71618574	Fn1	rs30571288	G	C*	splice_region_variant intron_variant _
1	71620826	Fn1	rs30574937	A	G*	splice_region_variant intron_variant _
1	71590092	Fn1	rs30565848	G	T*	splice_region_variant intron_variant downstream_gene_variant _
1	71629658	Fn1	rs30568781	C	G*	splice_region_variant intron_variant nmd_transcript_variant
1	71597274	Fn1	rs240078381	C	A*	splice_region_variant intron_variant nmd_transcript_variant downstream_gene_variant _
1	71597275	Fn1	rs258961698	A	C*	splice_region_variant intron_variant nmd_transcript_variant downstream_gene_variant _
1	71599595	Fn1	rs213492811	A	G*	splice_region_variant intron_variant nmd_transcript_variant downstream_gene_variant _
1	71600579	Fn1	rs30568853	A	G*	splice_region_variant intron_variant nmd_transcript_variant upstream_gene_variant downstream_gene_variant _
1	71601295	Fn1	rs243890686	G	A*	splice_region_variant intron_variant nmd_transcript_variant upstream_gene_variant downstream_gene_variant _
1	71599690	Fn1	rs228010915	A	G*	splice_region_variant synonymous_variant 3_prime_utr_variant nmd_transcript_variant downstream_gene_variant _
1	71615424	Fn1	rs30575238	G	A*	synonymous_variant _
1	71618640	Fn1	rs30571291	A	G*	synonymous_variant _
1	71619538	Fn1	rs226507739	C	A*	synonymous_variant _
1	71624328	Fn1	rs30574138	A	G*	synonymous_variant _
1	71629721	Fn1	rs13475914	G	C*	synonymous_variant 3_prime_utr_variant nmd_transcript_variant
1	71590167	Fn1	rs30565851	A	G*	synonymous_variant 3_prime_utr_variant nmd_transcript_variant _
1	71590795	Fn1	rs30566861	A	G*	synonymous_variant 3_prime_utr_variant nmd_transcript_variant _
1	71599255	Fn1	rs233491683	G	A*	synonymous_variant 3_prime_utr_variant nmd_transcript_variant downstream_gene_variant _
1	71599381	Fn1	rs30566152	T	C*	synonymous_variant 3_prime_utr_variant nmd_transcript_variant downstream_gene_variant _
1	71593156	Fn1	-	C	T*	synonymous_variant 3_prime_utr_variant nmd_transcript_variant upstream_gene_variant _
1	71600429	Fn1	rs227929578	A	T*	synonymous_variant 3_prime_utr_variant nmd_transcript_variant upstream_gene_variant downstream_gene_variant _
1	71629386	Fn1	rs30568775	G	A*	synonymous_variant downstream_gene_variant
1	71638572	Fn1	rs30570663	A	G*	synonymous_variant intron_variant nmd_transcript_variant downstream_gene_variant

Disease Models & Mechanisms • Supplementary information

1	71601280	Fn1	-	A	G*	synonymous_variant nmd_transcript_variant upstream_gene_variant downstream_gene_variant _
1	71605037	Fn1	rs235554628	G	A*	synonymous_variant upstream_gene_variant _
1	71605046	Fn1	rs30570301	A	G*	synonymous_variant upstream_gene_variant _
1	71605922	Fn1	rs30564847	A	G*	synonymous_variant upstream_gene_variant _
1	71609678	Fn1	rs265745224	G	A*	synonymous_variant upstream_gene_variant _
1	71610963	Fn1	rs30572992	A	G*	synonymous_variant upstream_gene_variant _
1	71645653	Fn1	rs264754433	C	T*	synonymous_variant upstream_gene_variant _
1	71645674	Fn1	rs30569218	T	C*	synonymous_variant upstream_gene_variant _
1	71649201	Fn1	rs30575794	T	C*	synonymous_variant upstream_gene_variant _
1	71649315	Fn1	rs30575797	A	G*	synonymous_variant upstream_gene_variant _
1	71626119	Fn1	rs30571465	G	A*	synonymous_variant upstream_gene_variant downstream_gene_variant
1	71626227	Fn1	rs264184462	C	T*	synonymous_variant upstream_gene_variant downstream_gene_variant
1	71626236	Fn1	rs229665825	G	C*	synonymous_variant upstream_gene_variant downstream_gene_variant
1	71629344	Fn1	rs30576850	G	A*	synonymous_variant upstream_gene_variant downstream_gene_variant
1	71642061	Fn1	rs30575196	C	T*	synonymous_variant upstream_gene_variant downstream_gene_variant
1	71586009	Fn1	rs215000101	T	TGTC*	3_prime_utr_variant downstream_gene_variant _
1	71584905	Fn1	rs241004945	A	AATCAGG	downstream_gene_variant
1	71585194	Fn1	rs264839825	CACACACTTGTATGTATGT	C	downstream_gene_variant
1	71585316	Fn1	rs226973260	CA	C	downstream_gene_variant
1	71585492	Fn1	rs249474020	TA	T	downstream_gene_variant
1	71608461	Fn1	rs223910329	G	GC*	intron_variant _
1	71609225	Fn1	-	TGCGTACGCGTGCGTGTGTGTGTGTGCGCGTGTGTGCGC	T*	intron_variant _
1	71616010	Fn1	rs262119165;rs243403134	AT	ATTTT*	intron_variant _
1	71616101	Fn1	rs223944857	GGGT	G*	intron_variant _
1	71616577	Fn1	rs223114172	AGTCT	A*	intron_variant _
1	71616996	Fn1	rs225556164	G	GA*	intron_variant _
1	71617685	Fn1	rs242805265	AG	A*	intron_variant _
1	71617962	Fn1	rs216619756	G	GT*	intron_variant _
1	71617974	Fn1	-	C	CTGT*	intron_variant _
1	71618027	Fn1	rs230039105	AATTTATTT	A*	intron_variant _
1	71618527	Fn1	rs263117240	CG	C*	intron_variant _
1	71618854	Fn1	rs217800738	C	CCT*	intron_variant _
1	71620185	Fn1	rs244717681	CTCT	C*	intron_variant _
1	71620643	Fn1	rs211931229	T	TTA*	intron_variant _
1	71621231	Fn1	rs243179227	C	CAAAAAA*	intron_variant _
1	71621350	Fn1	-	TTGTTTTG	T*	intron_variant _
1	71621397	Fn1	-	GC	G*	intron_variant _
1	71622110	Fn1	rs226417620	G	GT*	intron_variant _
1	71622536	Fn1	rs239700996	A	ATTTG*	intron_variant _
1	71623011	Fn1	-	GTGTGGAAGA	G*	intron_variant _
1	71623375	Fn1	rs218356098	TTTTG	T*	intron_variant _
1	71624039	Fn1	rs231441507	C	CG*	intron_variant _
1	71646564	Fn1	rs213338304	A	ATC*	intron_variant _
1	71647377	Fn1	rs256644315;rs218631356	AT	A*	intron_variant _
1	71587724	Fn1	rs227467585;rs256272269	AAAC	AAACAAC*	intron_variant downstream_gene_variant _
1	71587993	Fn1	rs220956049	C	CTTTGG*	intron_variant downstream_gene_variant _
1	71588046	Fn1	rs252687806	AC	A*	intron_variant downstream_gene_variant _
1	71588251	Fn1	rs215444297	G	GC*	intron_variant downstream_gene_variant _
1	71589984	Fn1	rs235167193	CCTAA	C*	intron_variant downstream_gene_variant _
1	71629569	Fn1	rs232501264	A	AAAGGGGAATGAATGCAGGT	intron_variant nmd_transcript_variant
1	71629859	Fn1	rs224147935	GA	G*	intron_variant nmd_transcript_variant
1	71630634	Fn1	rs236150268;rs233898224	ATT	A*	intron_variant nmd_transcript_variant
1	71632790	Fn1	rs264618889	CAA	CA*	intron_variant nmd_transcript_variant
1	71632933	Fn1	rs226777033	AAG	A*	intron_variant nmd_transcript_variant
1	71633176	Fn1	rs243757620	A	AT*	intron_variant nmd_transcript_variant
1	71634359	Fn1	rs219958301;rs260265275	TA	T*	intron_variant nmd_transcript_variant
1	71634432	Fn1	rs247572890	T	TG*	intron_variant nmd_transcript_variant
1	71635394	Fn1	rs229713064;rs248856466	GGTTTTGTTTT	GGTTTTGTTTTGTTTTGTTTT*	intron_variant nmd_transcript_variant
1	71635448	Fn1	-	GT	G*	intron_variant nmd_transcript_variant
1	71636868	Fn1	rs257888077	A	AAC*	intron_variant nmd_transcript_variant
1	71636968	Fn1	rs233793966	TACACACACAC	T*	intron_variant nmd_transcript_variant
1	71637713	Fn1	rs227606890	TA	T*	intron_variant nmd_transcript_variant
1	71590345	Fn1	rs257274204	A	AC*	intron_variant nmd_transcript_variant _
1	71590352	Fn1	-	TGGTAGACTA	T*	intron_variant nmd_transcript_variant _
1	71590397	Fn1	-	TGG	T*	intron_variant nmd_transcript_variant _
1	71639210	Fn1	rs243829256	AG	A*	intron_variant nmd_transcript_variant downstream_gene_variant
1	71639399	Fn1	rs221883873	TGGTTG	T*	intron_variant nmd_transcript_variant downstream_gene_variant
1	71639854	Fn1	rs241299422	G	GA*	intron_variant nmd_transcript_variant downstream_gene_variant
1	71640271	Fn1	rs257030542	C	CT*	intron_variant nmd_transcript_variant downstream_gene_variant
1	71640515	Fn1	rs232727439	GC	G*	intron_variant nmd_transcript_variant downstream_gene_variant
1	71640989	Fn1	rs243008286	GGTAAAAT	G*	intron_variant nmd_transcript_variant downstream_gene_variant
1	71596168	Fn1	rs238008091	TAA	TA*	intron_variant nmd_transcript_variant downstream_gene_variant _
1	71596276	Fn1	rs240732653	GA	G*	intron_variant nmd_transcript_variant downstream_gene_variant _
1	71597916	Fn1	rs225377130	A	ATT*	intron_variant nmd_transcript_variant downstream_gene_variant _
1	71597954	Fn1	rs258742558	GAAA	GAA*	intron_variant nmd_transcript_variant downstream_gene_variant _
1	71598333	Fn1	rs265629256	G	GA*	intron_variant nmd_transcript_variant downstream_gene_variant _
1	71598971	Fn1	rs237143995	A	AG*	intron_variant nmd_transcript_variant downstream_gene_variant _
1	71599526	Fn1	rs252963086	CCTAA	C*	intron_variant nmd_transcript_variant downstream_gene_variant _
1	71599577	Fn1	-	A	ACTTC*	intron_variant nmd_transcript_variant downstream_gene_variant _
1	71600017	Fn1	rs238983918	T	TA*	intron_variant nmd_transcript_variant downstream_gene_variant _
1	71591538	Fn1	rs217454721	A	AAGTCTCT*	intron_variant nmd_transcript_variant upstream_gene_variant _
1	71591813	Fn1	rs214543212	C	CTGAAG*	intron_variant nmd_transcript_variant upstream_gene_variant _
1	71592051	Fn1	rs237479769	T	TA*	intron_variant nmd_transcript_variant upstream_gene_variant _

Disease Models & Mechanisms • Supplementary information

1	71592610	Fn1	rs262376767	A	ATG*	intron_variant nmd_transcript_variant upstream_gene_variant _
1	71593330	Fn1	rs222761565	T	TCAG*	intron_variant nmd_transcript_variant upstream_gene_variant _
1	71594033	Fn1	rs247510210	G	GAA*	intron_variant nmd_transcript_variant upstream_gene_variant downstream_gene_variant _
1	71595404	Fn1	-	C	CCA*	intron_variant nmd_transcript_variant upstream_gene_variant downstream_gene_variant _
1	71601407	Fn1	rs264653895	CA	C*	intron_variant nmd_transcript_variant upstream_gene_variant downstream_gene_variant _
1	71601418	Fn1	-	GT	G*	intron_variant nmd_transcript_variant upstream_gene_variant downstream_gene_variant _
1	71601560	Fn1	rs227184928;rs216200212	CTTTT	CTTT*	intron_variant nmd_transcript_variant upstream_gene_variant downstream_gene_variant _
1	71601624	Fn1	rs257923904	C	CA*	intron_variant nmd_transcript_variant upstream_gene_variant downstream_gene_variant _
1	71601653	Fn1	rs250365829	A	ATGACCTGTCTT*	intron_variant nmd_transcript_variant upstream_gene_variant downstream_gene_variant _
1	71601814	Fn1	rs222988787	CT	C*	intron_variant nmd_transcript_variant upstream_gene_variant downstream_gene_variant _
1	71601872	Fn1	rs246523422	AT	A*	intron_variant nmd_transcript_variant upstream_gene_variant downstream_gene_variant _
1	71604316	Fn1	rs262306396	TC	T*	intron_variant upstream_gene_variant _
1	71604346	Fn1	rs235759101	CT	C*	intron_variant upstream_gene_variant _
1	71605410	Fn1	rs224495929	GT	G*	intron_variant upstream_gene_variant _
1	71605466	Fn1	rs247880311	AC	A*	intron_variant upstream_gene_variant _
1	71605584	Fn1	rs218628660	ATTTC	AC*	intron_variant upstream_gene_variant _
1	71606498	Fn1	-	G	gaaaaaacaaaaaaaaaa*	intron_variant upstream_gene_variant _
1	71606687	Fn1	rs246383126	C	CAG*	intron_variant upstream_gene_variant _
1	71607000	Fn1	rs254069160	TGACA	T*	intron_variant upstream_gene_variant _
1	71609740	Fn1	rs231587849	AGGAT	A*	intron_variant upstream_gene_variant _
1	71610272	Fn1	rs248578187	C	CGA*	intron_variant upstream_gene_variant _
1	71610342	Fn1	rs214769753	ACTAAGACAAGAAAC	A*	intron_variant upstream_gene_variant _
1	71610635	Fn1	rs241412311	C	CAT*	intron_variant upstream_gene_variant _
1	71611049	Fn1	rs265090502	CTTGA	C*	intron_variant upstream_gene_variant _
1	71611548	Fn1	-	T	TTGGGGTC*	intron_variant upstream_gene_variant _
1	71611673	Fn1	rs228783605	T	TCCTTGCGG*	intron_variant upstream_gene_variant _
1	71611727	Fn1	rs229605971	C	CA*	intron_variant upstream_gene_variant _
1	71611978	Fn1	rs262816939	TC	T*	intron_variant upstream_gene_variant _
1	71612700	Fn1	rs220348167;rs231663176	TAA	taaaaaaaa/taaaaaaa*	intron_variant upstream_gene_variant _
1	71612922	Fn1	rs248676681	CT	C*	intron_variant upstream_gene_variant _
1	71613472	Fn1	rs262649284	TA	T*	intron_variant upstream_gene_variant _
1	71614473	Fn1	rs235167654	TA	T*	intron_variant upstream_gene_variant _
1	71645957	Fn1	rs233844168	TAA	T*	intron_variant upstream_gene_variant _
1	71646253	Fn1	rs254798942	GT	G*	intron_variant upstream_gene_variant _
1	71648385	Fn1	rs237822495	AT	A*	intron_variant upstream_gene_variant _
1	71648731	Fn1	rs263866817	T	TCA*	intron_variant upstream_gene_variant _
1	71649447	Fn1	rs216570216	AT	A*	intron_variant upstream_gene_variant _
1	71649652	Fn1	rs243260033	G	GTCC*	intron_variant upstream_gene_variant _
1	71649882	Fn1	rs216259452	GA	G*	intron_variant upstream_gene_variant _
1	71650366	Fn1	rs235278827	G	GAC*	intron_variant upstream_gene_variant _
1	71650712	Fn1	rs216953887	GAA	GA*	intron_variant upstream_gene_variant _
1	71651731	Fn1	rs229026057	CAACT	C*	intron_variant upstream_gene_variant _
1	71651854	Fn1	rs245313894	G	GT*	intron_variant upstream_gene_variant _
1	71652060	Fn1	rs231074776	GC	G*	intron_variant upstream_gene_variant _
1	71652245	Fn1	rs237584075	AAAAGG	A*	intron_variant upstream_gene_variant _
1	71652359	Fn1	rs249555287	ACCC	A*	intron_variant upstream_gene_variant _
1	71624656	Fn1	rs252803690	CTT	C*	intron_variant upstream_gene_variant downstream_gene_variant
1	71624931	Fn1	rs222274201	TTTC	T*	intron_variant upstream_gene_variant downstream_gene_variant
1	71625570	Fn1	rs241923838	AT	A*	intron_variant upstream_gene_variant downstream_gene_variant
1	71625657	Fn1	rs231043880	AAGG	A*	intron_variant upstream_gene_variant downstream_gene_variant
1	71626377	Fn1	rs259238788	T	TA*	intron_variant upstream_gene_variant downstream_gene_variant
1	71626855	Fn1	rs218878571	CA	C*	intron_variant upstream_gene_variant downstream_gene_variant
1	71626958	Fn1	rs248750622	TG	T*	intron_variant upstream_gene_variant downstream_gene_variant
1	71628229	Fn1	rs253959475	G	GT*	intron_variant upstream_gene_variant downstream_gene_variant
1	71628887	Fn1	rs219544110;rs212599413	TG	TGTGG*	intron_variant upstream_gene_variant downstream_gene_variant
1	71628908	Fn1	-	T	TGGG*	intron_variant upstream_gene_variant downstream_gene_variant
1	71641863	Fn1	rs224359991	A	AGGAC*	intron_variant upstream_gene_variant downstream_gene_variant
1	71643318	Fn1	rs265453726	C	CA*	intron_variant upstream_gene_variant downstream_gene_variant
1	71643580	Fn1	rs223138229	C	CTG*	intron_variant upstream_gene_variant downstream_gene_variant
1	71643713	Fn1	rs252128985	GTCGGTGC	G*	intron_variant upstream_gene_variant downstream_gene_variant
1	71644273	Fn1	rs214474415	A	AG*	intron_variant upstream_gene_variant downstream_gene_variant
1	71602662	Fn1	rs230164235	A	AT*	intron_variant upstream_gene_variant downstream_gene_variant _
1	71602798	Fn1	rs212899566	A	AT*	intron_variant upstream_gene_variant downstream_gene_variant _
1	71603483	Fn1	rs221884930	C	CT*	intron_variant upstream_gene_variant downstream_gene_variant _
1	71645168	Fn1	rs228178818	TCTCA	T*	intron_variant upstream_gene_variant downstream_gene_variant _
1	71645526	Fn1	rs256012164	T	TC*	intron_variant upstream_gene_variant downstream_gene_variant _
1	71608024	Fn1	rs223349197	G	GA*	splice_region_variant intron_variant _
1	71644447-71644639	Fn1	-	-	-	deletion
1	71631167-71631202	Fn1	-	-	-	insertion

Disease Models & Mechanisms • Supplementary information

Table S4. Primer sequences for quantitative PCR.

Official Symbol	Forward Primer (5' to 3')	Reverse Primer (5' to 3')
Erbb4	AAGGCTACATGACTCCAATG	GCGAAGGTGTTGAGGTATAG
Ikzf2	GAGAGACCTGCTGTCATAGA	GGGTAGCTGAATCGCATAAG
Atic	GAGATCTCCAATGCGATTGAT	GGTCCACATTATCCCTGAAAG
Fn1	GACAACGGTGTCAACTACAA	TAGGTCTTCCCATCGTCATAG
Mreg	TAAAGCTGGCTGAAGAGACC	CTTGGAAATGGAAGGCAGTG
Pecr	GGTCAGAGCTACCTAGCGG	GACCACGTTACACCCCAGG
Tmem169	ATGAGAAGACGGATGAGGAG	TCCCGGTTAGTGTGACATAG
Xrcc5	TGGAAGTGTGAATCCTGTTG	GCTTTGATGCAGTCCATACT
March4	GCTATGGGATGTATGGCTTC	CACTTTCCACTGCTGGTTTA
Smarcal1	ATGACGGAAGCTACAGACTA	GTTTCCAGAGGTTCCTGAAG
Rpl37a	GCTAAACGCACCAAGAAGGT[illegible]	GCCACTGTTTTCATGCAGGAA
Igfbp2	CAGACGCTACGCTGCTATCC	CCCTCAGAGTGGTCGTCATCA
Igfbp5	AGACAGGAATCCGAACAAGG[illegible]	GTAGAATCCTTTGCGGTCACA
Hprt1	TGACACTGGCAAAACAATGCA	GGTCCTTTTCACCAGCAAGCT
Gapdh	AGGTCGGTGTGAACGGATTTG	TGTAGACCATGTAGTTGAGGTCA

Disease Models & Mechanisms • Supplementary information
